# A three-classification model for identifying migraine with right-to-left shunt using lateralization of functional connectivity and brain network topology: a resting-state fMRI study

**DOI:** 10.3389/fnins.2024.1488193

**Published:** 2024-11-12

**Authors:** Weifang Nie, Weiming Zeng, Jiajun Yang, Lei Wang, Yuhu Shi

**Affiliations:** ^1^Lab of Digital Image and Intelligent Computation, Shanghai Maritime University, Shanghai, China; ^2^Department of Neurology, Shanghai Sixth People’s Hospital, Shanghai, China

**Keywords:** laterality index, fMRI, right-to-left shunt, migraine, classification

## Abstract

**Introduction:**

Right-to-left shunting has been significantly associated with migraine, although the neural mechanisms remain complex and not fully elucidated. The aim of this study was to investigate the variability of brain asymmetry in individuals with migraine with right-to-left shunting, migraine without right-to-left shunting and normal controls using resting-state fMRI technology and to construct a three-classification model.

**Methods:**

Firstly, asymmetries in functional connectivity and brain network topology were quantified to laterality indices. Secondly, the laterality indices were employed to construct a three-classification model using decision tree and random forest algorithms. Ultimately, through a feature score analysis, the key brain regions that contributed significantly to the classification were extracted, and the associations between these brain regions and clinical features were investigated.

**Results:**

Our experimental results showed that the initial classification accuracy reached 0.8961. Subsequently, validation using an independent sample set resulted in a classification accuracy of 0.8874. Further, after expanding the samples by the segmentation strategy, the classification accuracies were improved to 0.9103 and 0.9099. Additionally, the third sample set yielded a classification accuracy of 0.8745. Finally, 9 pivotal brain regions were identified and distributed in the default network, the control network, the visual network, the limbic network, the somatomotor network and the salience/ventral attention network.

**Discussion:**

The results revealed distinct lateralization features in the brains of the three groups, which were closely linked to migraine and right-to-left shunting symptoms and could serve as potential imaging biomarkers for clinical diagnosis. Our findings enhanced our understanding of migraine and right-to-left shunting mechanisms and offered insights into assisting clinical diagnosis.

## 1 Introduction

Migraine, a prevalent chronic neurological disorder, was characterized by periodic episodes of severe headaches, usually accompanied by nausea, vomiting, and photophobia (Schwedt et al., 2015). According to the Global Burden of Disease study 2019, the global age-standardized prevalence and annual incidence of migraine were 14,107.3 and 11,425.5 per 100,000 people, respectively (Safiri et al., 2022). The disease ranked second among global causes of disability, particularly occupying the first place among young women (Steiner et al., 2020). Its pathogenesis was quite complex and its key etiological factors had not been fully revealed so far. Specifically, the relationship between migraine and right-to-left shunt (RLS) had emerged as a focal point of research. The prevalence of RLS in adults varied between 10 and 35% (Teshome et al., 2020), in which patent foramen ovale (PFO) were the most common type (Küper et al., 2013; Dang et al., 2021). Recent research indicated that the prevalence of PFO among migraine with aura (MWA) patients ranged from 40 to 60% (Kumar et al., 2019), and the prevalence of MWA was higher in patients with moderate to large shunts (Wilmshurst, 2018). Furthermore, multiple studies had shown that intervening to close RLS could reduce migraine symptoms, suggesting a potentially involvement of RLS in the underlying mechanisms that trigger migraine attacks (Wahl et al., 2010; Ling et al., 2020). Despite some progress in existing studies, further investigation was required to elucidate the precise connection between migraine and RLS and the role of RLS in the pathogenesis of migraine.

Functional magnetic resonance imaging (fMRI) had opened up new avenues for exploring the deeper pathological mechanisms of migraine due to its non-invasive nature, feasibility for repeated testing, ease of use, and high spatial resolution (Ogawa et al., 1990). At the level of large-scale brain networks, studies revealed significant differences in the dynamic functional connectivity and global topological properties between migraine patients and healthy controls (Shi et al., 2020). In addition, pain intensity during migraine attacks has been associated with weakened connectivity between the default mode network (DMN) and the insula (Coppola et al., 2018). At the level of the brain region, the dynamic functional connectome technique revealed that the key brain regions involved in migraine included Brodmann areas 1/2/3, the basal ganglia, and the thalamus, which were closely related to pain. Additionally, the occipital lobe was identified as a key region directly associated with migraine symptoms (Nie et al., 2021). It was evident that fMRI could help to explore the neural mechanisms of migraine. Nevertheless, for RLS and migraine, initial neuroimaging studies focused on white matter hyperintensity (Cao et al., 2022). Additionally, three principal techniques were employed in the diagnosis of RLS: transthoracic echocardiography, transesophageal echocardiography, and transcranial Doppler. It should be noted that there was no diagnostic method based on fMRI data. Consequently, there was a paucity of fMRI studies on neurological mechanisms and imaging diagnosis, which were worthy of further investigation by researchers.

The study of asymmetry in brain structure and function had a long history and was an important research focus in the field of neuroscience. The subtle structural differences and varying levels of dominance in the left and right hemispheres during specific tasks were termed hemispheric lateralization (Duboc et al., 2015). Several studies showed that the left hemisphere had stronger activation in positive emotional processing, while the right hemisphere had more intense activation in negative emotional processing (Waldstein et al., 2000; Güntürkün et al., 2020; Beraha et al., 2012). Furthermore, it had also been shown that there was also a hemispheric asymmetry in attention, with the right side of the brain dominating in the control of attention, especially in the control of attentional redirection (Asanowicz et al., 2012). However, in the research on the relationship between migraine and RLS, there are almost no relevant research results. There is still insufficient research on the changes of lateralization in migraine and RLS, as well as how to use brain asymmetry to assist doctors in diagnosis and treatment.

In this study it was hypothesized that brain asymmetry differs among individuals with migraine with RLS (the RLS group), migraine without RLS (the NRLS group) and normal individuals. Moreover, these differences were postulated to have the potential to be employed in the diagnosis of the disease. To test these hypotheses, this study analyzed brain asymmetry at the level of brain regions and constructed a three-classification model based on it. In section 2, laterality indices of brain regions were constructed based on functional connectivity and topological properties of brain networks. Subsequently, a three-classification model was constructed based on the lateralization indices, employing the decision tree and random forest algorithms to differentiate among the RLS group, NRLS group, and normal group. Subsequently, an independent dataset was employed for a validation test. Then, with an increase in the sample size achieved through a segmentation strategy, the classification process was executed once more for the purpose of testing the validation. Ultimately, the key brain regions were extracted based on classification accuracy and feature importance scores, and the correlations between these brain regions and clinical features were investigated. Section 3 presented the experimental results, while section 4 offered discussions. Finally, conclusions were drawn in section 5.

## 2 Materials and methods

### 2.1 Data acquisition

Raw resting-state functional magnetic resonance imaging (RS-fMRI) data were acquired from migraine patients at the Neurology Department of Shanghai Jiao Tong University Affiliated Sixth People’s Hospital, utilizing a 3 T Siemens scanner (Erlangen, Germany). The imaging parameters included the acquisition of 38 slices covering all brain regions, with a repetition time (TR) of 3.0 s, a total of 160 time points and the voxel size of 1x1x1 mm. The study enrolled 27 migraineurs (23 females, 4 males; mean age 39.70 ± 11.03 years) diagnosed with chronic migraine according to the International Classification of Headache Disorders, 3rd Edition (Olesen, 2018). These patients were categorized into two groups: 11 individuals exhibiting a right-to-left shunt during resting state (RLS group) and 16 without right-to-left shunt (NRLS group), based on the presence of embolic signals detected through a contrast-enhanced transcranial doppler (cTCD) assessment during a resting-state foaming experiment. Ethical approval for the study was granted by the Independent Ethics Committee of Shanghai Sixth People’s Hospital East Campus (Ethical No. 2019–016). The mean ages for the RLS and NRLS groups were 37.09 and 41.50 years, respectively (Table 1). Two-sample t-tests were used to examine potential differences in demographics, including gender, age, education level, illness duration, attack frequency, and headache severity (measured by the visual analog scale, VAS), between the two groups. No statistically significant differences were found between the RLS and NRLS cohorts in these characteristics, as detailed in Table 1. To control for age and gender, and following the exclusion criteria outlined in Section 2.2, we obtained additional RS-fMRI data from 16 healthy individuals (12 females, 4 males; mean age 36.19 ± 12.83 years) from a publicly available database,^1^ provided by Alan C. Evans and referred to as ICBM. This dataset consisted of 23 slices covering the entire brain, a TR of 2.0 s, 128 time points and the voxel size of 1x1x1 mm. Normality and homogeneity of variance tests were conducted for age and gender. The Kruskal-Wallis H-test corrections yielded a *p*-value of 0.41 for age and the chi-squared test resulted in a p-value of 0.2738 for gender, indicating no significant age differences between the healthy controls and the migraineurs, and confirming that the control group was well-matched with the migraineurs in terms of gender and age. Additionally, RS-fMRI data from another 16 healthy individuals (12 females, 4 males; mean age 33.69 ± 9.33 years) were accessed from the Center for Biomedical Research Excellence (named COBRE).^2^ The scanning parameters for this dataset included 33 slices covering the entire brain, a TR of 2.0 s, 150 time points and the voxel size of 1x1x1 mm. Using the similar statistical testing method to ICBM dataset, the age *p*-value of 0.1178 and the gender p-value of 0.2738 further confirmed that this dataset was also appropriately matched with the migraineurs in terms of gender and age. Furthermore, another 5 healthy individuals (3 females, 2 males; mean age 25 ± 0.7017 years) were sourced from a publicly accessible database (see Footnote 1), provided by Yufeng Zhang from China and cited as Zhang_Beijing. The dataset comprised 33 slices encompassing the entire brain, a TR of 2.0 s, 225 time points and the voxel size of 1x1x1 mm.

**Table 1:** Characteristics and clinical profiles for patients.

	Total	RLS group	NRLS group	*P* value
Gender (Male/Female)	4/23	3/8	1/15	0.1412
Age	39.70 ± 11.03	37.09 ± 7.60	41.50 ± 12.89	0. 3,170
Level of education attainment (year)	13.52 ± 3.65	14.64 ± 4.31	12.75 ± 2.11	0. 1927
Duration of illness (years)	16.65 ± 10.67	13.7 ± 7.87	18.5 ± 11.97	0. 2,732
Frequency of attacks (times/month)	2.66 ± 2.94	2.79 ± 2.90	2.58 ± 3.05	0.7329
VAS	8.34 ± 1.16	8.30 ± 1.05	8.37 ± 1.24	0.8759

VAS, visual analog score. All values were expressed as mean ± standard deviation.

### 2.2 Data preprocessing

The RS-fMRI data were subjected to a series of preprocessing steps using the Data Processing Assistant (Yan and Zang, 2010). The steps included: (1) the initial 10 time points were discarded to reduce the impact of T1 equilibration effects; (2) slice timing correction was performed with the central slice as the reference; (3) participants with excessive head movement, defined as more than 2.0 mm of displacement or 1.5° of rotation, were excluded to reduce motion-related artifacts, and the remaining data underwent realignment for motion correction; (4) the data were spatially normalized to the echo-planar imaging template from the Montreal Neurological Institute; and (5) Gaussian smoothing was applied using a 6 mm kernel.

### 2.3 The construction of quantitative index of asymmetry

**Figure 1:**
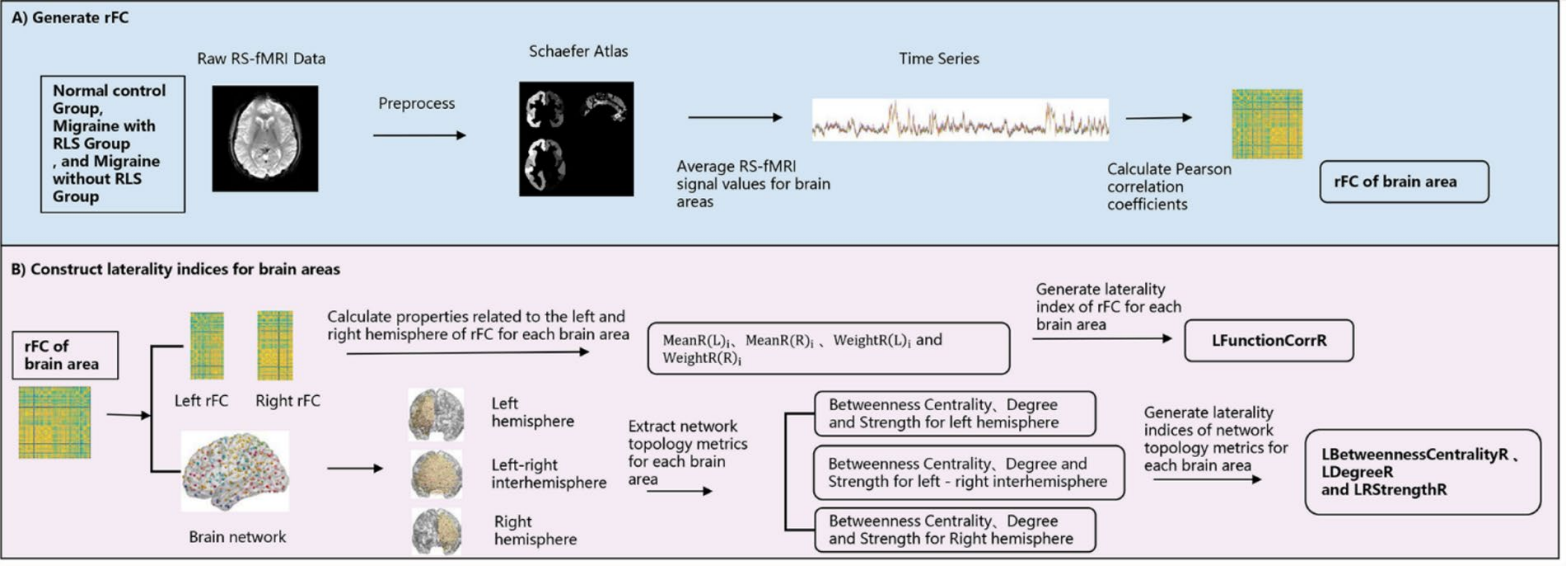
Schematic framework for the construction of the laterality indices. **(A)** The generation process of region-to-region functional connectivity; **(B)** the construction process of laterality indices.

The primary objective of this section was to compute quantitative measures of asymmetry across all brain regions by leveraging functional connectivity and brain network topology. The research methodology was detailed in Figure 1. As illustrated in Figure 1A, the Schaefer 2018 atlas (refer to Supplementary Figure S1; Schaefer et al., 2018; Yeo et al., 2011) was utilized to segment the RS-fMRI data into 400 distinct regions of interest (ROIs). The time series for each ROI were obtained by averaging the RS-fMRI signal values within that region. Subsequently, we calculated the Pearson correlation coefficient between each ROI and a target brain area, generating region-to-region functional connectivity (rFC) maps. Given the ongoing debate about the validity of negative connections, which may be influenced by various factors such as network anticorrelations, global signal regression, and phase-shifting soft tissue correction, we opted to include only positive functional connections, setting negative connections to zero. Figure 1B offered a detailed depiction of the construction of laterality indices based on both functional connectivity and brain network topology.

#### 2.3.1 The construction of laterality index based on functional connectivity

In previous studies on lateralization quantification (Liu et al., 2009; Gotts et al., 2013), the following formula was used.

(1) $$\mathrm{Laterality}\;\mathrm{Inde}x_{i}=\frac{X{\left(L\right)}_{i}-X{\left(R\right)}_{i}}{X{\left(L\right)}_{i}+X{\left(R\right)}_{i}}$$

(2) $$\mathrm{Segregatio}n_{i}=X{\left(LL\right)}_{i}-X{\left(LR\right)}_{i}-\left(X{\left(RR\right)}_{i}-X{\left(RL\right)}_{i}\right)$$

(3) $$\mathrm{Integratio}n_{i}=X{\left(LL\right)}_{i}+X{\left(LR\right)}_{i}-\left(X{\left(RR\right)}_{i}+X{\left(RL\right)}_{i}\right)$$

In Equation 1, $X{\left(L\right)}_{i}$ was used to denote the characteristics of the *ith* ROI in the left hemisphere, while $X{\left(R\right)}_{i}$ was employed to represent the characteristics of the *ith* ROI in the right hemisphere. In Equation 2, the initial letter in each label denoted the seed hemisphere, while the second letter denoted the target hemisphere. For example, $X{\left(LR\right)}_{i}$ signified the correlation between a seed node in the left hemisphere and all target surface nodes in the right hemisphere. A positive value for $X{\left(LL\right)}_{i}-X{\left(LR\right)}_{i}$ indicated that the average relationship of this ROI within the left hemisphere was stronger than the average relationship with right hemispheres. In Equation 3, a positive value of the integration metric indicated a greater degree of bilateral interaction with nodes in the left hemisphere, whereas a negative value suggests a stronger bilateral interaction with nodes in the right hemisphere. The three formulas utilized brain templates with aligned left and right hemisphere positions to delineate brain regions. However, as numerous brain functions did not adhere rigidly to a symmetric distribution, calculations based on the aligned positioning of the left and right hemispheres might potentially overlook some local or functional differences. Furthermore, these formulas calculated a single lateralization value from the ROIs corresponding to the left and right hemispheres, which might not fully reflect the functional lateralization pattern of the actual brain. This resulted in the potential for more fine-grained regional information to be overlooked.

In order to improve the previous formulas, this paper proposed an approach that combined the advantages of the previous formulas and quantified the lateralization value of the functional connectivity of each ROI by introducing weighting coefficients. Specifically, the formula is as follows:

(4) $$\mathrm{LFunctionCorr}R_{i}=\left(\frac{\begin{array}{l} \mathrm{MeanR}{\left(L\right)}_{i}*\mathrm{WeightR}{\left(L\right)}_{i}-\\ \mathrm{MeanR}{\left(R\right)}_{i}*\mathrm{WeightR}{\left(R\right)}_{i} \end{array}}{\begin{array}{l} \mathrm{MeanR}{\left(L\right)}_{i}*\mathrm{WeightR}{\left(L\right)}_{i}+\\ \mathrm{MeanR}{\left(R\right)}_{i}*\mathrm{WeightR}{\left(R\right)}_{i} \end{array}}\right)$$

In this context, $\mathrm{MeanR}{\left(L\right)}_{i}$ represented the mean rFC strength between the *ith* ROI and the left hemisphere ROIs, calculated as $\mathrm{MeanR}{\left(L\right)}_{i}=\left(\overset{200}{\underset{j=1}{\sum }}Corr_{i,j}\right)/n$ . Similarly, $\mathrm{MeanR}{\left(R\right)}_{i}$ represented the mean rFC strength between the *ith* ROI and the right hemisphere ROIs, calculated as $\mathrm{MeanR}{\left(R\right)}_{i}=\left(\overset{400}{\underset{j=201}{\sum }}Corr_{i,j}\right)/m\text{.}$ The weighting coefficients $\mathrm{WeightR}{\left(L\right)}_{i}$ and $\mathrm{WeightR}{\left(R\right)}_{i}$ represented the weights of rFC strength of the ROI in the left and right hemispheres, specifically $\mathrm{WeightR}{\left(L\right)}_{i}$ = $\mathrm{MeanR}{\left(L\right)}_{i}$ / $\mathrm{mean}\left(\mathrm{MeanR}\left(L\right)\right)$ and $\mathrm{WeightR}{\left(R\right)}_{i}$ = $\mathrm{MeanR}{\left(R\right)}_{i}$ / $\mathrm{mean}\left(\mathrm{MeanR}\left(R\right)\right)$ (1 ≤ i ≤ 200).

The formula was based on $X{\left(LL\right)}_{i}-X{\left(LR\right)}_{i}$ and $X{\left(RR\right)}_{i}-X{\left(RL\right)}_{i}$ from Equation 2 in order to measure the difference in rFC strength between the left and right hemispheres. In particular, the $X{\left(LL\right)}_{i}$ of ROIs in the left hemisphere and $X{\left(RL\right)}_{i}$ of ROIs in the right hemisphere were simplified as $\mathrm{MeanR}{\left(L\right)}_{i}$ , signifying the mean rFC strength between the node and the nodes in the left hemisphere. Similarly, the $X{\left(LR\right)}_{i}$ of ROIs in the left hemisphere and $X{\left(RR\right)}_{i}$ of ROIs in the right hemisphere were simplified to $\mathrm{MeanR}{\left(R\right)}_{i}$ , representing the mean rFC strength between the node and the nodes in the right hemisphere. By comparing the inner hemisphere connections ( $X{\left(LL\right)}_{i}$ and $X{\left(RR\right)}_{i}$ ) and cross-hemisphere connections ( $X{\left(LR\right)}_{i}$ and $X{\left(RL\right)}_{i}$ ) of each region, it was possible to calculate the degree of lateralization of the region toward the left or right hemisphere in functional connections. This method allowed for the capture of differences in interaction strength between each region and its own hemisphere or the opposite hemisphere in terms of functional connectivity, thereby providing a different perspective from that offered by traditional lateralization analysis. Furthermore, the formula employed the concept of division as seen in Equation 1, limiting the lateralization value to the range of −1 to 1, thereby enhancing the clarity and comparability of the degree of lateralization. To enhance the precision of the analysis, the formula incorporates weighting coefficients to accentuate regions that were pivotal in functional connectivity and attenuate regions that exert less influence on functional connectivity. The weighting coefficients reflected the relative importance of each region in the left and right hemispheres, thereby avoiding the introduction of noise or bias into the lateralization analysis results caused by weakly connected regions. By introducing the weights in a reasonable manner, it was possible to more effectively highlight the regions that played a pivotal role in the functional network. This formula had some advantages in functional template analysis, particularly in the context of the Schaefer 2018 brain atlas utilized in this article, where the localization of the left and right hemisphere regions was not entirely aligned. In comparison to the constraints of aligning left and right hemisphere regions in previous studies, this formula could more effectively reflect the functional asymmetry between the left and right hemispheres of the brain through a more flexible and precise lateralization quantification method.

#### 2.3.2 The construction of laterality indices based on brain network topological properties

In order to construct the lateralization for the network topology attributes, the following formulae were employed, which were based on the approach set out in Equation 4:

(5) $$\mathrm{LNetworkMR}{\left(X\right)}_{i}=\left(\frac{\begin{array}{l} XR{\left(L\right)}_{i}*\mathrm{WeigtR}{\left(L\right)}_{i}-\\ XR{\left(R\right)}_{i}*\mathrm{WeigtR}{\left(R\right)}_{i} \end{array}}{\begin{array}{l} XR{\left(L\right)}_{i}*\mathrm{WeigtR}{\left(L\right)}_{i}+\\ XR{\left(R\right)}_{i}*\mathrm{WeigtR}{\left(R\right)}_{i} \end{array}}\right)$$

Firstly, two networks representing the left and right hemispheres (200 × 200) respectively, as well as a network representing inter-hemispheric connections (400 × 400) were constructed based on rFCs. Then three network properties—betweenness centrality, degree, and strength—were utilized and were described in Supplementary Table S1. These properties revealed the centrality, connectivity, and connection strength of nodes in the brain network, providing crucial clues for understanding the organizational structure and functional mechanisms of brain networks. To ensure the reliability and consistency of the study, the two hemisphere networks and one inter-hemisphere network of each participant were thresholded. The threshold range was set from 0.6 to 0.95 to cover different strengths of connectivity patterns. To mitigate the impact of threshold selection randomness, we further calculated the area under the curve (AUC) for each metric (Achard and Bullmore, 2007). Then for nodes in the left hemisphere, $XR{\left(L\right)}_{i}$ was defined as the network property of ROI nodes in the left hemisphere, while $XR{\left(R\right)}_{i}$ represented the network property of ROI nodes between the left and right hemispheres. Conversely, when dealing with nodes in the right hemisphere, $XR{\left(L\right)}_{i}$ was considered as representing the properties of ROI nodes between the left and right hemispheres, while $XR{\left(R\right)}_{i}$ represented the network properties of ROI nodes in the right hemisphere. To further quantify the influence of each ROI node, specific weighting coefficients were introduced, namely $\mathrm{WeigtR}{\left(L\right)}_{i}$ = $XR{\left(L\right)}_{i}$ /mean (*XR(L)*) and $\mathrm{WeigtR}{\left(R\right)}_{i}$ = $XR{\left(R\right)}_{i}$ /mean (*XR(R)*). Using Equation 5, laterality indices were computed for 400 brain areas. Similarly, the range of these indices was from −1 to 1, with positive values indicating a leftward bias and negative values indicating a rightward bias in the attributes. For the three network properties, corresponding laterality indices were LBetweennessCentralityR, LDegreeR, and LStrengthR.

### 2.4 The three-classification model based on laterality indices

#### 2.4.1 The construction of the three-classification model and the evaluation of classification performance

**Figure 2:**
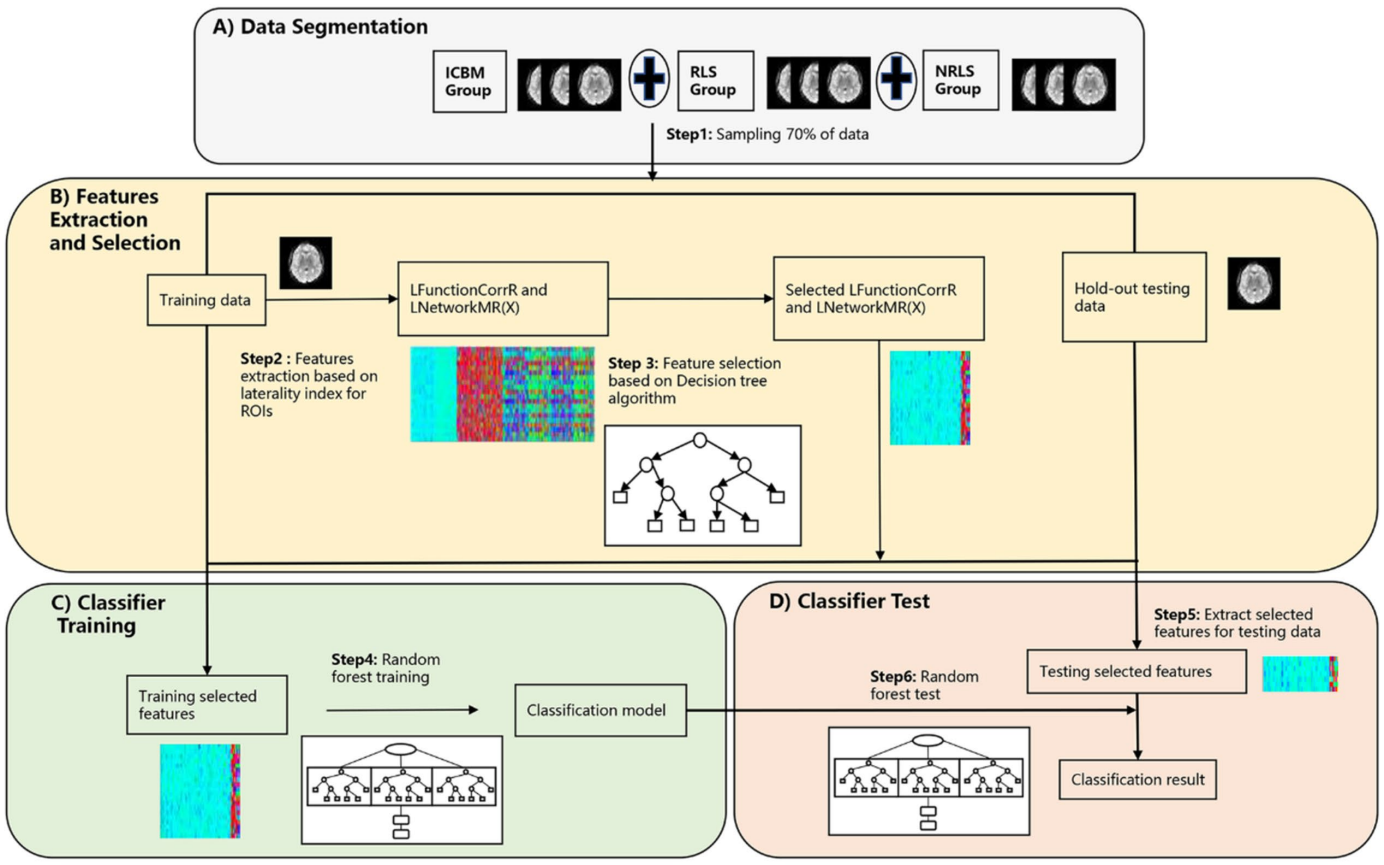
The procedure of the three-classification model based on laterality indices. **(A)** The segmentation process of preprocessed RS-fMRI data; **(B)** the feature extraction and selection process based on laterality indices; **(C)** the classifier training process using training data; **(D)** the testing process of testing data.

The model adopted in this section was depicted in Figure 2, which detailed the comprehensive steps. Initially, a random selection process was utilized to designate 70% of the participants from three groups for feature selection and classification model training, with the remaining 30% set aside for later classification testing, as outlined in Figure 2A. The LFunctionCorrR values were then computed using Equation 4, and the LNetworkMR(X) values were obtained using Equation 5. Each participant provided 400*4 features, with the specific extraction process detailed in Figure 2B. To refine the accuracy of feature selection, decision tree algorithms were incorporated for filtering and refining the features, as shown in Figure 2B. Subsequently, the logistic regression model was utilized to recalibrate the class weights, thereby addressing the issue of data imbalance within the dataset. Then a grid search was conducted to identify the optimal parameters for the random forest classifier. Next, a random forest classifier was subsequently developed using these refined features, as illustrated in Figure 2C. After the classifier was trained, the optimized features were extracted from the reserved test set, as shown in Figure 2D. The trained classifier was then applied to classify the participants in the test set. To confirm the stability and dependability of this method, the entire sequence was executed 100 times, and the mean classification performance was determined, as illustrated in Figure 2D. Subsequently, a TR division was applied for each participant, splitting those with 150 TR into two 75 TR subjects for migraines and those with 118 TR into two 59 TR subjects for ICBM, to expand the sample size. Ultimately, the classification was conducted once more following the procedures outlined in Figure 2.

#### 2.4.2 The evaluation of the validity/reproducibility of the three-classification model

**Figure 3:**
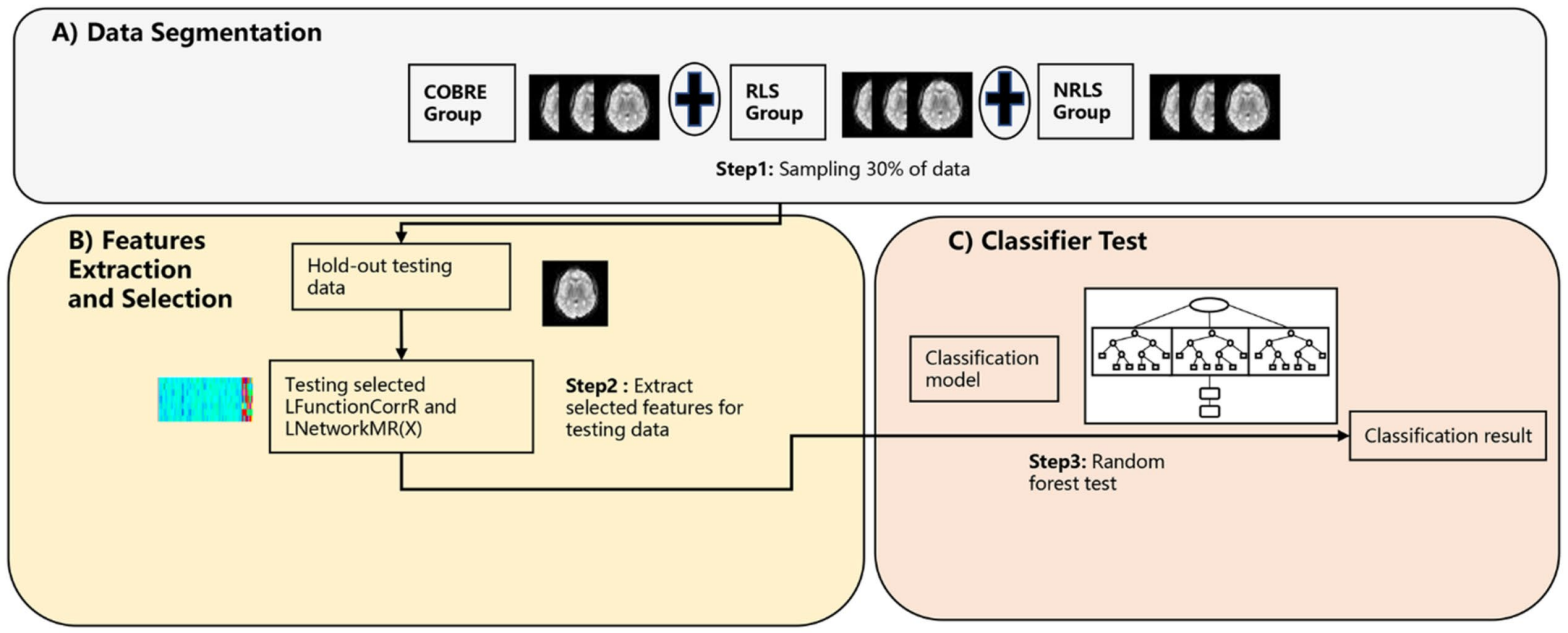
The procedure of validity/repeatability testing. **(A)** The segmentation process of preprocessed RS-fMRI data; **(B)** the feature extraction and selection process based on laterality indices; **(C)** the testing process of testing data.

The purpose of this section is to authenticate the three-classification model proposed in 2.4.1 and to ascertain the reproducibility and generalization of classification model based on these features across diverse participant group (refer to Figure 3). To achieve this objective, the investigation was extended to included normal subjects from a different data center abbreviated as COBRE (refer to Figure 3A). The subjects underwent the same processing protocols as outlined in 2.4.1. Following processing, we extracted the refined LFunctionCorrR and LNetworkMR(X) features, which were previously selected based on the remaining 30% of data from the RLS group and NRLS group in section 2.4.1 and a comparable 30% from the COBRE group (refer to Figures 3A,B). Subsequently, the classifier trained in section 2.4.1 was employed to distinguish between RLS, NRLS, and normal subjects (refer to Figure 3C). Notably, no subjects from this study participated in any model fitting stages, encompassing feature extraction, parameter tuning, or model training. Additionally, employing the TR segmentation approach, the COBRE subject with 140 TR were divided into two subjects 70 TR to enhance sample size. Finally, the classification process was repeated, adhering to the procedures depicted in Figure 3.

To further validate the reliability of the algorithm, this study selected 5 healthy subjects from China and matched them with 5 subjects from the RLS and NRLS groups, respectively. The RLS group consisted of 3 females and 2 males, with an average age of 31.2 years (standard deviation 2.77), while the NRLS group included 4 females and 1 male, with an average age of 27.4 years (standard deviation 5.31). A Kruskal-Wallis H test was employed to ascertain whether there were any significant differences in age between the two groups. The resulting *p*-value of 0.059 indicated that there was no statistically significant difference in age distribution between the two groups. Gender distribution was analyzed using a chi-square test, with a p-value of 0.7559, showing no significant gender differences between the groups. Due to the limited sample size, the study employed a data segmentation strategy to increase the dataset and enhance the robustness of the analysis. This approach involved dividing each subject’s data into five parts. Subsequently, the algorithm was subjected to a re-evaluation in accordance with the procedure delineated in Figure 2. The segmentation method effectively expanded the sample size to verify the reliability and stability of the algorithm’s testing results.

### 2.5 The identification of crucial brain region

The lateralization feature scores were introduced to quantify how each feature contributes to the classification process. This metric was formulated by integrating the classification accuracy with and the feature importance values obtained from random forest analysis. The method for calculating this score was detailed below:

(6) $$\mathrm{LScor}e_{i}=\mathrm{FeatureImportance}*\frac{\left(\mathrm{Accurary}1_{i}+\mathrm{Accurary}2_{i}\right)}{2}$$

The FeatureImportance score, assigned by the random forest algorithm, quantified the significance of each feature and its contribution to the classification task. The Accurary1 and Accurary2 evaluated the effectiveness of the features in the identification process. Features that were excluded from a given classification received a score of zero. The cumulative score for each feature was calculated by summing its scores across 100 classification iterations [i.e., ( $\mathrm{LScor}e_{i}$ )].For each region of interest (ROI), a total of 400 × 4 feature scores were derived. By examining the top 40 features ranked for the brain areas, it became viable to accurately detect specific brain regions that exhibit a significant association with RLS.

The number of embolic signals detected in the intracranial arteries during the foaming test was used to categorize RLS grades. These grades are classified as follows: minimal right-to-left shunt (1–10 embolic signals), moderate right-to-left shunt (11–25 embolic signals), and large right-to-left shunt (more than 25 embolic signals) (Ailani, 2014). For the NRLS group, we examined the correlation (*p* < 0.05) between the laterality indices of brain regions and factors such as disease duration, attack frequency, and headache scores. For the RLS group, we further analyzed (*p* < 0.05) the relationships between the laterality indices and disease duration, attack frequency, headache scores, and RLS grades.

## 3 Results

### 3.1 Results of laterality indices

**Figure 4:**
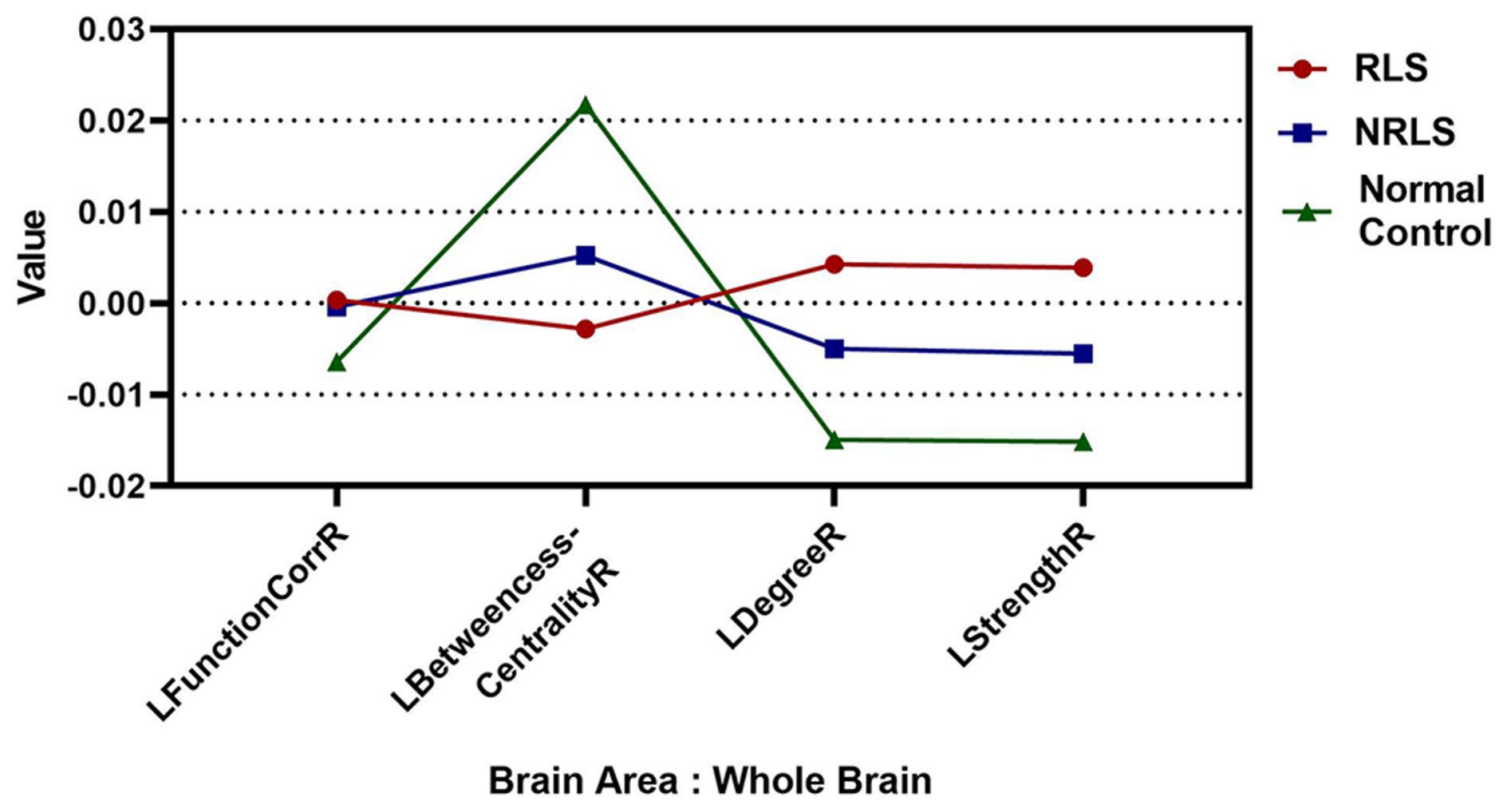
The mean values of Laterality indices at the whole brain level for three groups.

In this study, the values for LFunctionCorrR and LNetworkMR(X) were calculated for the 400 ROIs using Equations 4, 5. Figure 4 illustrated the distribution of LFunctionCorrR and LNetworkMR(X) across the whole brain for the three participant groups. The mean value of LFunctionCorrR was 0.0004 for the RLS group, −0.0004 for the NRLS group, and − 0.0064 for the normal control group. Regarding the network properties of brain regions, the mean values of LBetweennessCentralityR, LDegreeR, and LStrengthR for the RLS group were −0.0028, 0.0042, and 0.0039, respectively. For the NRLS group, these values were 0.0052, −0.0049, and −0.0055, respectively, while for the normal control group, they were 0.0217, −0.0149, and −0.0152, respectively.

**Figure 5:**
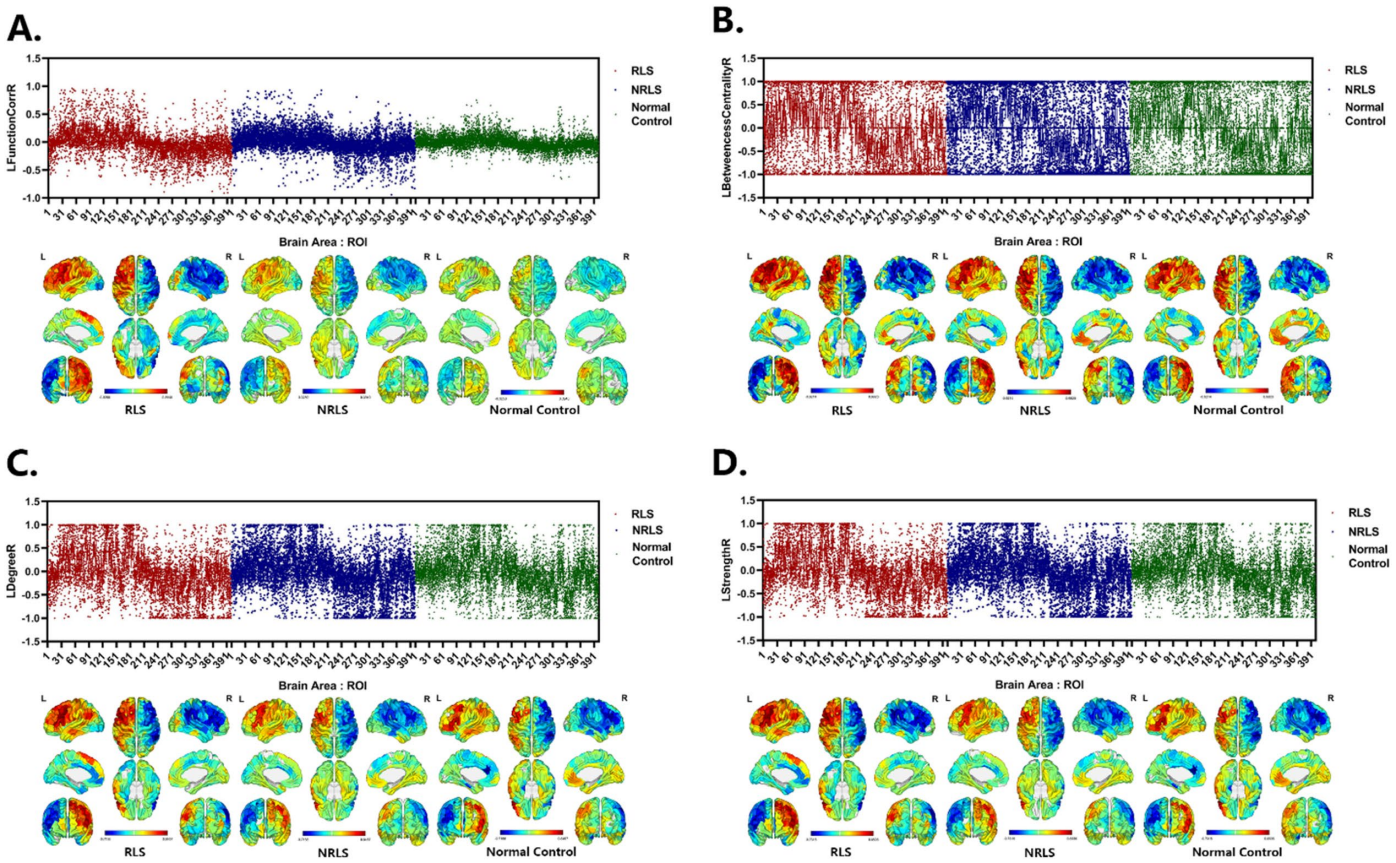
Laterality indices of brain areas for three groups. (A–D) LFunctionCorrR and LNetworkMR(X) of ROIs for the three groups. Upper: the mean with SEM for LFunctionCorrR and LNetworkMR(X) of ROIs. Lower: visualization of mean LFunctionCorrR and LNetworkMR(X) of ROIs projected onto a standard brain surface.

Figures 5A–D illustrated the distribution of LFunctionCorrR and LNetworkMR(X) across brain regions for the three participant groups. This figure displayed the average values and standard errors (SEM) of LFunctionCorrR and LNetworkMR(X) for 400 regions of interest (ROIs). The data were projected onto a standard brain surface, providing a clear visualization of the average values of LFunctionCorrR and LNetworkMR(X) for each brain region.

### 3.2 The three-classification model results

**Figure 6:**
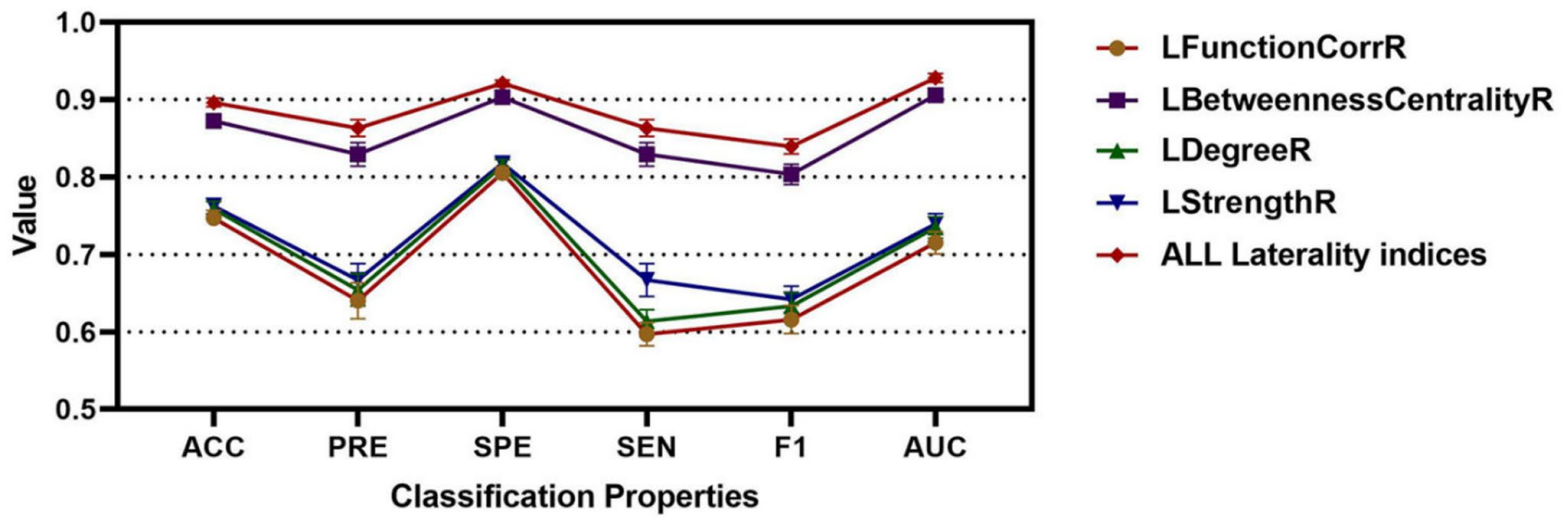
The classification accuracy, precision, specificity, sensitivity, F1, and AUC for different features for RLS, NRLS and ICBM groups.

In this study, feature sets were derived from the laterality indices of brain regions to distinguish between the three participant groups. Four feature sets, labeled as LFunctionCorrR and LNetworkMR(X), were obtained from the brain regions for classification purposes. The classification outcomes were shown in Table 2, providing the average performance metrics for each classification feature. Additionally, Figure 6 displayed the average accuracy, precision, specificity, sensitivity, F1 score, and area under the ROC curve for the various features, each with their 95% confidence intervals. Supplementary Figure S2A displayed the ROC curve for the ICBM dataset when all features were utilized.

**Table 2:** Performance evaluation of classification model with different features for ICBM dataset.

Features	ACC	PRE	SPE	SEN	F1	ROC
LFunctionCorrR	0.7470	0.6401	0.8054	0.5969	0.6157	0.8054
LBetweenness-CentralityR	0.8723	0.8292	0.9031	0.7796	0.8034	0.9031
LDegreeR	0.7586	0.6541	0.8148	0.6137	0.6333	0.7349
LStrengthR	0.7625	0.6669	0.8176	0.6193	0.6418	0.7394
LFunctionCorrR + LBetweennessCentralityR + LDegreeR + LStrengthR	0.8961	0.8630	0.9209	0.8185	0.8395	0.9280

ACC, accuracy; PRE, precision; SPE, specificity; SEN, sensitivity; AUC, area under the ROC curve.

In order to provide a comprehensive evaluation of the performance of the algorithm proposed in this paper, we adopted the methods proposed by Liu et al. (2009) and Gotts et al. (2013) for calculating the laterality indices based on the Schaefer 2023 symmetric atlas (Yan et al., 2023). Subsequently, these values were applied to the classification task for three groups of subjects, with the aim of comparing the results with those obtained by the algorithm in this paper. To further investigate the efficacy of the algorithms, an additional set of experiments was designed to compare the role of weighting coefficients in the algorithms by removing the weighting coefficients to calculate the laterality indices and classifying the same three groups of subjects again. These comparison experiments were applied on the same subject dataset, and their classification performances were recorded. The results of these comparisons were presented in detail in Table 3 and Supplementary Figure S3.

**Table 3:** Performance evaluation of classifier with different approaches for ICBM.

Approach	ACC	PRE	SPE	SEN	F1	ROC
Liu et al. (2009)	0.7428	0.6406	0.8002	0.5873	0.6137	0.7011
Gotts et al. (2013)	0.7625	0.6807	0.8158	0.6174	0.6494	0.7353
LFunctionCorrR + LBetweennessCentralityR + LDegreeR + LStrengthR without weighting coefficients	0.8674	0.8093	0.8994	0.7701	0.7891	0.9048
LFunctionCorrR + LBetweennessCentralityR + LDegreeR + LStrengthR with weighting coefficients	0.8961	0.8630	0.9209	0.8185	0.8395	0.9280

ACC, accuracy; PRE, precision; SPE, specificity; SEN, sensitivity; AUC, area under the ROC curve.

**Figure 7:**
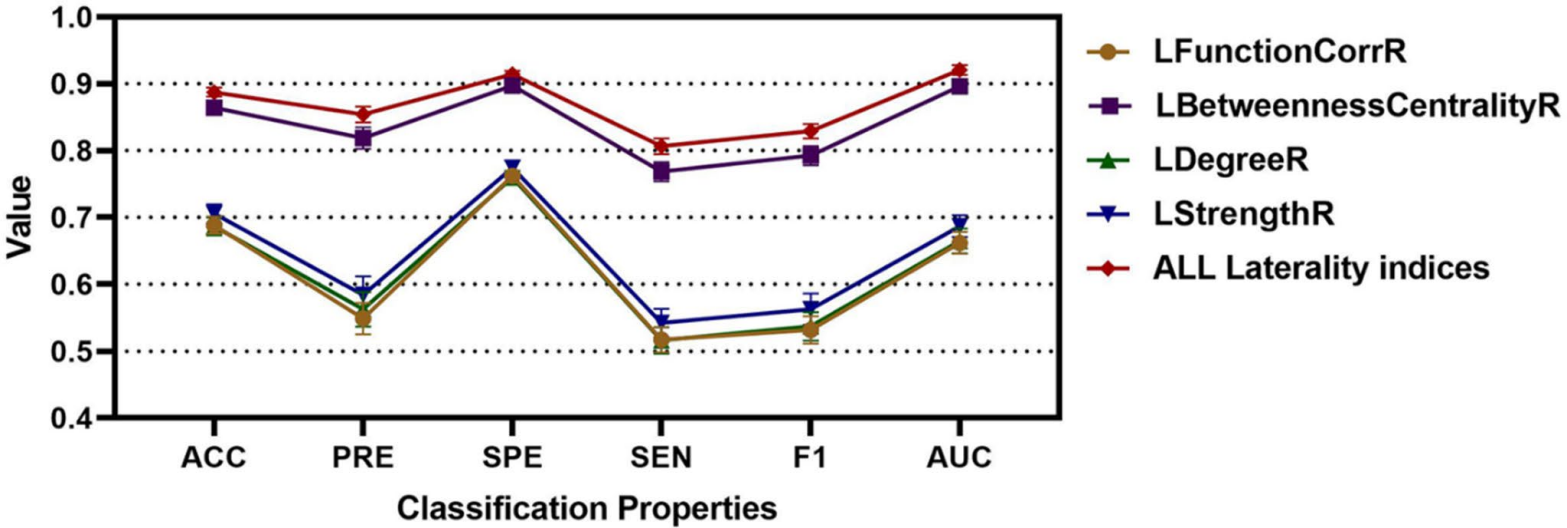
The classification accuracy, precision, specificity, sensitivity, F1, and AUC for different features for RLS, NRLS and COBRE groups.

To substantiate the effectiveness and repeatability of the proposed method, this study employed a separate dataset known as COBRE for testing. The findings were summarized in Table 4 and visually depicted in Figure 7. Table 4 displayed the average classification performance for each feature, while Figure 7 illustrated the average values along with 95% confidence intervals for key metrics such as accuracy, precision, specificity, sensitivity, F1 score, and the ROC curve, assessed across different features. Table 5 and Supplementary Figure S4 presented a comparison of the experimental results of this paper with those of the methods of Liu et al. (2009), Gotts et al. (2013), and without weighting coefficients. Supplementary Figure S2B presented the ROC curve for the COBRE dataset when all features were employed.

**Table 4:** Performance evaluation of classification model with different features for COBRE dataset.

Features	ACC	PRE	SPE	SEN	F1	ROC
LFunctionCorrR	0.6884	0.5487	0.7624	0.5173	0.5318	0.7624
LBetweenness-CentralityR	0.8642	0.8186	0.8970	0.7687	0.7925	0.8964
LDegreeR	0.6867	0.5626	0.7596	0.5161	0.5370	0.6647
LStrengthR	0.7056	0.5843	0.7744	0.5422	0.5628	0.6869
LFunctionCorrR + LBetweennessCentralityR + LDegreeR + LStrengthR	0.8874	0.8541	0.9141	0.8066	0.8290	0.9205

ACC, accuracy; PRE, precision; SPE, specificity; SEN, sensitivity; AUC, area under the ROC curve.

**Table 5:** Performance evaluation of classifier with different approaches for COBRE.

Approach	ACC	PRE	SPE	SEN	F1	ROC
Liu et al. (2009)	0.6740	0.5321	0.7482	0.4940	0.5117	0.7482
Gotts et al. (2013)	0.6874	0.5441	0.7594	0.5155	0.5263	0.7594
LFunctionCorrR + LBetweennessCentralityR + LDegreeR + LStrengthR without weighting coefficients	0.8574	0.8009	0.8920	0.7568	0.7781	0.8949
LFunctionCorrR + LBetweennessCentralityR + LDegreeR + LStrengthR with weighting coefficients	0.8874	0.8541	0.9141	0.8066	0.8290	0.9205

ACC, accuracy; PRE, precision; SPE, specificity; SEN, sensitivity; AUC, area under the ROC curve.

**Figure 8:**
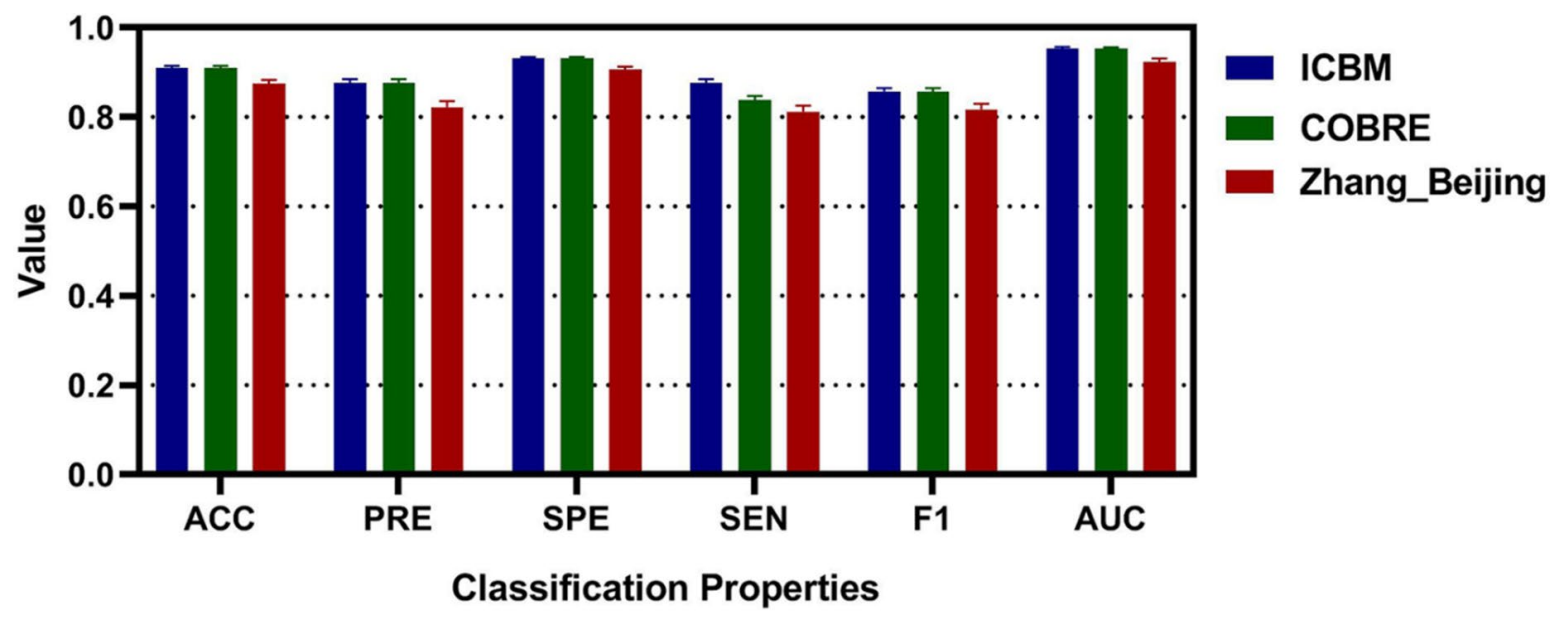
The classification accuracy, precision, specificity, sensitivity, F1, and AUC for different dataset after segmenting.

To enhance the validation of the proposed methods’ accuracy, this study augmented the sample size by segmenting the RS-fMRI data of patients. These segmented samples were then analyzed for classification using the laterality indices of brain regions. Moreover, to ascertain the suitability and generalization of the algorithm proposed in this study, an additional Zhang_Beijing dataset was incorporated as a test subject. Due to the relatively limited data sample size of this dataset, an approach was employed whereby the data of one subject was split into five parts, thus simulating a larger sample of subjects and achieving the effect of increasing the sample size. The average performance metrics of the three datasets were shown in Table 6. Additionally, Figure 8 presented the classification performance metrics of the different datasets, along with their 95% confidence intervals, offering a detailed visual overview for evaluating classification effectiveness. Supplementary Figures S2C–E illustrated the ROC curve for all features after segmentation.

**Table 6:** Performance evaluation of classification model after segmenting.

Dataset	ACC	PRE	SPE	SEN	F1	ROC
ICBM	0.9103	0.8764	0.9313	0.8387	0.8567	0.9534
COBRE	0.9099	0.8763	0.9309	0.8381	0.8564	0.9529
Zhang_Beijing	0.8745	0.8221	0.9061	0.8124	0.8171	0.9235

ACC, accuracy; PRE, precision; SPE, specificity; SEN, sensitivity; AUC, area under the ROC curve.

### 3.3 Crucial brain areas extracted based on feature scores

**Figure 9:**
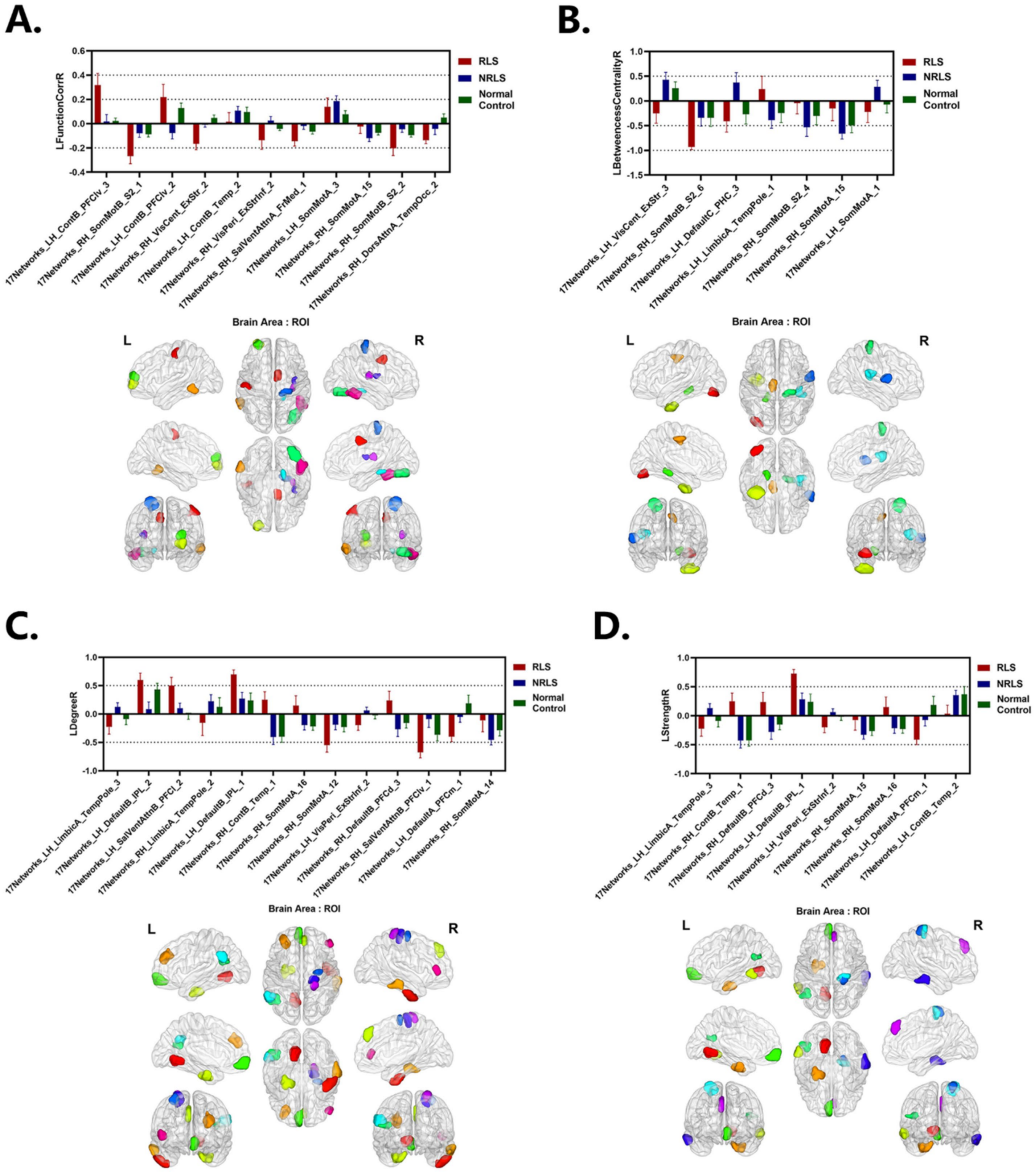
Laterality indices of the selected ROIs for each group. (A–D) LFunctionCorrR and LNetworkMR(X) of the selected ROIs with top 40 features score. Upper: the mean with SEM for LFunctionCorrR and LNetworkMR(X) of the selected ROIs. Lower: ROIs selected projected onto a standard brain surface.

In this study, the Equation 6 was utilized to compute the feature scores of LFunctionCorrR and LNetworkMR(X) for the brain regions. Table 7 provided a comprehensive list of the top 40 brain regions along with their feature scores. Furthermore, Figure 9 visually presented the information of LFunctionCorrR and LNetworkMR(X) for the selected brain regions. From the data in Table 7, it was observed that the brain region ROI238 (named 17Networks_RH_SomMotA_15) appeared three times across the four features, while the regions ROI14 (named 17Networks_LH_VisPeri_ExStrInf_2), ROI116 (named 17Networks_LH_LimbicA_TempPole_3), ROI135 (named 17Networks_LH_ContB_Temp_2), ROI161 (named 17Networks_LH_DefaultA_PFCm_1), ROI173 (named 17Networks_LH_DefaultB_IPL_1), ROI239 (named 17Networks_RH_SomMotA_16), ROI336 (named 17Networks_RH_ContB_Temp_1), and ROI379 (named 17Networks_RH_DefaultB_PFCd_3) appeared twice. Therefore, brain regions exhibiting asymmetrical changes for RLS and migraine encompassed ROI14 (named 17Networks_LH_VisPeri_ExStrInf_2), ROI116 (named 17Networks_LH_LimbicA_TempPole_3), ROI135 (named 17Networks_LH_ContB_Temp_2), ROI161 (named 17Networks_LH_DefaultA_PFCm_1), ROI173 (named 17Networks_LH_DefaultB_IPL_1), ROI238 (named 17Networks_RH_SomMotA_15), ROI239 (named 17Networks_RH_SomMotA_16), ROI336 (named 17Networks_RH_ContB_Temp_1), and ROI379 (named 17Networks_RH_DefaultB_PFCd_3).

**Table 7:** The (ROI)* information for top 40 features score.

Features	ROI number	ROI name	Score
LFunctionCorrR	142	17Networks_LH_ContB_PFClv_3	1.7264
	247	17Networks_RH_SomMotB_S2_1	1.3565
	141	17Networks_LH_ContB_PFClv_2	1.0716
	202	17Networks_RH_VisCent_ExStr_2	0.8601
	135	17Networks_LH_ContB_Temp_2	0.8327
	214	17Networks_RH_VisPeri_ExStrInf_2	0.6181
	296	17Networks_RH_SalVentAttnA_FrMed_1	0.5476
	27	17Networks_LH_SomMotA_3	0.5246
	238	17Networks_RH_SomMotA_15	0.5245
	248	17Networks_RH_SomMotB_S2_2	0.4667
	260	17Networks_RH_DorsAttnA_TempOcc_2	0.4587
LBetweenness CentralityR	3	17Networks_LH_VisCent_ExStr_3	3.2555
	253	17Networks_RH_SomMotB_S2_6	0.9084
	194	17Networks_LH_DefaultC_PHC_3	0.8993
	114	17Networks_LH_LimbicA_TempPole_1	0.8909
	251	17Networks_RH_SomMotB_S2_4	0.6140
	238	17Networks_RH_SomMotA_15	0.5774
	25	17Networks_LH_SomMotA_1	0.5479
LDegreeR	116	17Networks_LH_LimbicA_TempPole_3	3.3489
	174	17Networks_LH_DefaultB_IPL_2	1.7879
	102	17Networks_LH_SalVentAttnB_PFCl_2	1.6415
	320	17Networks_RH_LimbicA_TempPole_2	1.1336
	173	17Networks_LH_DefaultB_IPL_1	0.9373
	336	17Networks_RH_ContB_Temp_1	0.8544
	239	17Networks_RH_SomMotA_16	0.6139
	235	17Networks_RH_SomMotA_12	0.5771
	14	17Networks_LH_VisPeri_ExStrInf_2	0.5367
	379	17Networks_RH_DefaultB_PFCd_3	0.5315
	305	17Networks_RH_SalVentAttnB_PFClv_1	0.5228
	161	17Networks_LH_DefaultA_PFCm_1	0.5200
	237	17Networks_RH_SomMotA_14	0.4673
LStrengthR	116	17Networks_LH_LimbicA_TempPole_3	3.4086
	336	17Networks_RH_ContB_Temp_1	2.5139
	379	17Networks_RH_DefaultB_PFCd_3	1.2896
	173	17Networks_LH_DefaultB_IPL_1	1.2728
	14	17Networks_LH_VisPeri_ExStrInf_2	0.8061
	238	17Networks_RH_SomMotA_15	0.7600
	239	17Networks_RH_SomMotA_16	0.7409
	161	17Networks_LH_DefaultA_PFCm_1	0.6164
	135	17Networks_LH_ContB_Temp_2	0.5499

According to the correlation analysis, it was revealed whether there existed correlations between the lateralization of the extracted brain regions and the clinical characteristics of RLS and NRLS patients. The *p*-values yielded the following results. It was found that significant correlations were observed in the NRLS group between the duration of illness and laterality indices of multiple ROIs, including a negative correlation with LFunctionCorrR of 17Networks_LH_ContB_Temp_2 (correlation coefficient c = −0.5373, significance level *p* = 0.0319), and a positive correlation with LFunctionCorrR and LStrengthR of 17Networks_RH_SomMotA_15 (LFunctionCorrR: c = 0.5031, *p* = 0.0470; LStrengthR: c = 0.5162, *p* = 0.0407). In the RLS group, the duration of illness was positively correlated with LBetweennessCentralityR of 17Networks_RH_SomMotA_15 (c = 0.7397, *p* = 0.0145) in the RLS group. Furthermore, the VAS of RLS was positively correlated with LFunctionCorrR of 17Networks_LH_ContB_Temp_2 (c = 0.6338, *p* = 0.0491), LDegreeR of 17Networks_RH_SomMotA_16 (c = 0.6608, *p* = 0.0375), and LStrengthR of 17Networks_RH_SomMotA_16 (c = 0.6725, *p* = 0.0331). It is noteworthy that there was a significant negative correlation between the severity grading of RLS (RLS grading) and LStrengthR of 17Networks_LH_ContB_Temp_2 (c = −0.7587, *p* = 0.0110). These findings provided new clues for understanding the neural mechanisms of RLS. For detailed information, please refer to Supplementary Tables S2, S3.

## 4 Discussion

### 4.1 Laterality indices for brain areas

This study explored the lateralization phenomena of functional connectivity and network properties in brain regions. To quantify this, four laterality indices were introduced: LFunctionCorrR, LBetweennessCentralityR, LDegreeR, and LStrengthR. According to Figure 4, when analyzing the brain, the NRLS group and the normal control group exhibited right-lateralization in LFunctionCorrR, LDegreeR, and LStrengthR, but left-lateralization in LBetweennessCentralityR. It was noteworthy that the RLS group demonstrated the opposite pattern: left-lateralization in LFunctionCorrR, LDegreeR, and LStrengthR, and right-lateralization in LBetweennessCentralityR. This reversed pattern of lateralization implies that the two groups of patients might use different brain region activation strategies when processing brain tasks. This difference might stem from the effects brought about by RLS.

As could be seen in visualization of mean LFunctionCorrR and LNetworkMR(X) of Figure 5, for the level of the specific brain area, most of the left hemisphere of the subjects in three groups showed a tendency to lateralize to the left, while most of the right hemisphere was lateralized to the right, and this phenomenon was consistent across all four attributes. Notably, the RLS group showed higher values for LFunctionCorrR in compared to the NRLS group and the normal control group. This suggested that the RLS group might have experienced more pronounced lateralization, likely associated with their specific pathophysiological mechanisms. Specifically, RLS might have caused the concentration of certain functions in specific hemispheres, which could have led to abnormalities in brain function and an imbalance in processing related functions. This functional imbalance could have potentially triggered or exacerbated migraine-related symptoms.

### 4.2 Classification model performance

This study employed the laterality indices of brain regions as discriminative attributes for classification. Initially, using the ICBM dataset as the control group, these laterality indices were integrated with decision tree feature extraction and a random forest classifier. The results showed that for the LFunctionCorrR feature, the average metrics were 0.7470 for accuracy, 0.6401 for precision, 0.8054 for specificity, 0.5969 for sensitivity, 0.6157 for the F1 score, and 0.8054 for the ROC value. For the LBetweennessCentralityR feature, the averages were 0.8723 in accuracy, 0.8292 in precision, 0.9031 in specificity, 0.7796 in sensitivity, 0.8034 in F1 score, and 0.9031 in ROC value. The LDegreeR feature yielded averages of 0.7586 for accuracy, 0.6541 for precision, 0.8148 for specificity, 0.6137 for sensitivity, 0.6333 for F1 score, and 0.7349 for ROC value. The LStrengthR feature provided averages of 0.7625 for accuracy, 0.6669 for precision, 0.8176 for specificity, 0.6193 for sensitivity, 0.6418 for F1 score, and 0.7394 for ROC value. When all four features were combined, the averages improved to 0.8961 for accuracy, 0.8630 for precision, 0.9209 for specificity, 0.8185 for sensitivity, 0.8395 for F1 score, and 0.9280 for ROC value. According to Table 2 and Figure 6, the using of LFunctionCorrR and LNetworkMR(X) features resulted in better classification performance compared to using any single feature type, indicating that these features together provided the best results.

Consistent findings were observed with an independent dataset, COBRE. For this dataset, the LFunctionCorrR feature had average metrics of 0.6884 for accuracy, 0.5487 for precision, 0.7624 for specificity, 0.5173 for sensitivity, 0.5318 for F1 score, and 0.7624 for ROC value. The LBetweennessCentralityR feature achieved averages of 0.8642 in accuracy, 0.8186 in precision, 0.8970 in specificity, 0.7687 in sensitivity, 0.7925 in F1 score, and 0.8970 in ROC value. For the LDegreeR feature, the averages were 0.6867 for accuracy, 0.5626 for precision, 0.7596 for specificity, 0.5161 for sensitivity, 0.5370 for F1 score, and 0.6647 for ROC value. The LStrengthR feature produced averages of 0.7056 for accuracy, 0.5843 for precision, 0.7744 for specificity, 0.5422 for sensitivity, 0.5628 for F1 score, and 0.6869 for ROC value. When combining the LFunctionCorrR and LNetworkMR(X) features, the results improved to 0.8874 in accuracy, 0.8541 in precision, 0.9141 in specificity, 0.8066 in sensitivity, 0.8290 in F1 score, and 0.9205 in ROC value. As shown in Table 4 and Figure 7, the model utilizing both LFunctionCorrR and LNetworkMR(X) features outperformed those using individual features, demonstrating that these features together provided the most reliable classification at the individual level and suggesting the model had good generalization ability.

Furthermore, comparative analyses with traditional algorithms (Liu et al., 2009; Gotts et al., 2013), were conducted in order to verify the effectiveness of the proposed method. As evidenced in Tables 3, 5, the proposed method in this study exhibited notable superiority in classification performance. In particular, when ICBM was employed as the control group, our method attained the highest values for all classification metrics, including a classification accuracy of 0.8961. Moreover, in the classification task for the independent sample set COBRE, the algorithm proposed in this study demonstrated superior performance compared to the algorithm proposed by Liu et al. (2009) and Gotts et al. (2013). In particular, our method achieved a classification accuracy of 0.8874 on the COBRE dataset. These findings not only underscored the superiority of the present study’s method in capturing lateralized features of brain function, but also suggested a potential unique association between migraine-associated RLS disease patterns and asymmetric changes in the brain. These findings had implications for a deeper understanding of migraine pathomechanisms and the development of targeted diagnostic and therapeutic strategies. In order to provide a more robust assessment of the methodology employed in this study, further comparisons were made between the classification effects of laterality indices with weighting coefficients and without weighting coefficients. As demonstrated in Tables 3, 5, the classification effect of laterality indices with weighting coefficients was superior to that of laterality indices with without weighting coefficients, across both datasets. The results demonstrated the efficacy of the weighting coefficients proposed in this study in capturing the lateralization in each brain region. The introduction of these weighting coefficients allowed for a more accurate quantification of the contribution of different brain regions in the process of lateralization, thereby elucidating the intricate mechanisms underlying brain function lateralization.

Finally, to confirm the reproducibility and reliability of the model, this study employed a strategy of segmenting the subjects, thereby expanding the sample size. Furthermore, a third dataset, the Zhang_Beijing dataset, comprising exclusively Chinese individuals, was incorporated. When using the ICBM dataset for classification, the results showed an average accuracy of 0.9103, precision of 0.8764, specificity of 0.9313, sensitivity of 0.8387, F1 score of 0.8567, and ROC value of 0.9534. For the COBRE dataset, the average metrics were similarly effective, with accuracy at 0.9099, precision at 0.8763, specificity at 0.9309, sensitivity at 0.8381, F1 score at 0.8564, and ROC value at 0.9529. The final validation of the model was carried out using the Zhang_Beijing dataset. The model’s performance on this dataset was consistent with that observed on the previous two datasets. The average metrics, including accuracy of 0.8745, precision of 0.8221, specificity of 0.9061, sensitivity of 0.8124, F1 score of 0.8171, and ROC value of 0.9235. These results from Table 6 and Figure 8 further highlighted the effectiveness of laterality indices in classification tasks and their potential to capture variations in brain function in this three-classification model, which offered valuable evidences for early diagnosis and a deeper understanding of functional differences among migraineurs with and without RLS, and normal subjects.

### 4.3 Crucial brain areas with asymmetrical changes for migraine and RLS

In this study, the top 40 ranked lateralized attribute values were extracted from LFunctionCorrR and LNetworkMR(X) by the feature score value method, and the associated brain regions were identified. Finally, the brain regions ROI14 (named 17Networks_LH_VisPeri_ExStrInf_2), ROI116 (named 17Networks_LH_LimbicA_TempPole_3), ROI135 (named 17Networks_LH_ContB_Temp_2), ROI161 (named 17Networks_LH_DefaultA_PFCm_1), ROI173 (named 17Networks_LH_DefaultB_IPL_1), ROI238 (named 17Networks_RH_SomMotA_15), ROI239 (named 17Networks_RH_SomMotA_16), ROI336 (named 17Networks_RH_ContB_Temp_1), and ROI379 (named 17Networks_RH_DefaultB_PFCd_3) were identified as those most closely associated with migraine and RLS.

It was noted that 17Networks_LH_VisPeri_ExStrInf_2 (which was in extra-striate inferior of peripheral visual), a brain region within the visual network, exhibited left lateralization in LDegreeR and LStrengthR in the NRLS group, while right lateralization was observed in the RLS group and normal control group, with a more pronounced degree of lateralization in the RLS group (see Figure 9). This indicated that the connectivity and strength of this brain region were more inclined toward the left hemisphere for NRLS subjects, whereas it was more biased toward the right hemisphere for RLS subjects, and more concentrated in RLS subjects. Previous studies revealed abnormalities in the structure, microstructure, and functional connectivity of the extra-striate cortex in migraine patients, indicating that the extra-striate cortex was involved in the origin of visual auras (Russo et al., 2019; Russo et al., 2018; Silvestro et al., 2022). In comparison to healthy controls, migraine patients exhibited a higher frequency of activation of the contralateral extra-striate visual cortex (Schwedt et al., 2015; Vincent et al., 2003). These studies are consistent with our findings, suggesting that that RLS might cause changes in the functional connectivity and strength of visual-related brain regions through mechanisms such as microemboli or decreased blood oxygen saturation, resulting in differences in lateralization.

17Networks_LH_LimbicA_TempPole_3 was in the temporal pole (TP) of limbic A network. Figure 9 indicated that the NRLS group exhibited a left lateralization in both LDegreeR and LStrengthR in the 17Networks_LH_LimbicA_TempPole_3, whereas the RLS group and normal control group demonstrated a right lateralization. Furthermore, the magnitude of the lateralization in the RLS group was found to be greater than that observed in the NRLS group and normal control group. The TP played an important role in socio-emotional processes such as face recognition and theory of mind (Olson et al., 2007). Additionally, previous research indicated that there was a significant rightward advantage in the efficiency and betweenness of nodes in the limbic system (Sun et al., 2017; Caeyenberghs and Leemans, 2014; Shu et al., 2015; Wang et al., 2023), and that normal individuals also displayed a rightward asymmetry in node degree in the temporal pole median region (Wang et al., 2023; Li et al., 2021). Notably, migraine patients showed enhanced activation in the medial temporal lobe, particularly in the anterior temporal pole (TP), which might be related to functional abnormalities in migraine (Moulton et al., 2011). The results of this study indicated that RLS caused a change in the direction of the lateralization of the node degree and node strength in the 17Networks_LH_LimbicA_TempPole_3 with an increase in the magnitude of the lateralization. This suggested that RLS had a significant impact on the lateralization of this region, implying that RLS might induce hemispheric changes in the nodal properties of this area, which could be closely related to the emergence of associated neurological symptoms.

17Networks_LH_ContB_Temp_2 (which was in temporal of left control B network) was associated with RLS and migraine. Figure 9 illustrated that three groups exhibited left lateralization in LFunctionCorrR and LStrengthR in this brain region, with the NRLS group and healthy control group showing a greater degree of lateralization than the RLS group. Furthermore, Supplementary Tables S2, S3 indicated that LFunctionCorrR of 17Networks_LH_ContB_Temp_2 in the NRLS group was negatively correlated with disease duration, while LStrengthR of 17Networks_LH_ContB_Temp_2 in the RLS group was negatively correlated with RLS grading and positively correlated with VAS. This indicated that as the disease progressed, the lateralization of functional connectivity in 17Networks_LH_ContB_Temp_2 decreased, which may reflect the disruption of synchronization and coordination between the left and right hemispheres caused by the disease. Similarly, as the RLS grade increased, the lateralization of connection strength in 17Networks_LH_ContB_Temp_2 exhibited a gradual decline. And the increase in VAS could lead to an increase in lateralization of connection strength in 17Networks_LH_ContB_Temp_2. This might lead to cognitive impairments closely related to the severity of RLS symptoms. Another brain region, 17Networks_RH_ContB_Temp_1, which was in temporal of right control B network, showed that NRLS group and healthy control group exhibited right lateralization in LDegreeR and LStrengthR, whereas the RLS group showed the opposite. This indicated that 17Networks_RH_ContB_Temp_1 had higher connectivity and connection strength in the right hemisphere for the NRLS group and healthy control group, while the RLS group showed the reverse pattern. This might reflect the specific impact of RLS on the right hemisphere function of 17Networks_RH_ContB_Temp_1, potentially related to the neural mechanisms underlying the disease condition. These findings further confirmed the impact of RLS on the frontoparietal control network and suggested a potential link between this impact and migraine.

17Networks_LH_DefaultA_PFCm_1 was situated in the ventral medial prefrontal cortex of the left default A network. Figure 9 indicates that the LDegreeR and LStrengthR of 17Networks_LH_DefaultA_PFCm_1exhibited a rightward lateralization in both RLS and NRLS groups, and leftward shift in the normal group, with the largest magnitude in the RLS group. This change indicated that RLS might cause 17Networks_LH_DefaultA_PFCm_1to connect more strongly with other brain regions in the right hemisphere. This was consistent with earlier studies on the functional asymmetry of the mPFC, which had demonstrated a functional difference between the right and left hemispheres of the mPFC in emotion processing. The left hemisphere was found to be more associated with positive emotions, while the right hemisphere was more involved in the processing of negative emotions (Waldstein et al., 2000; Beraha et al., 2012). Meanwhile, some studies had shown a reduction in the functional connectivity of mPFC regions in migraine patients (Ke et al., 2020; Chen et al., 2022). In light of the negative emotions frequently experienced by migraine patients, it was postulated that the enhanced right lateralization of 17Networks_LH_DefaultA_PFCm_1 might be associated with the migraine symptoms and emotional consequences induced by RLS. Furthermore, 17Networks_LH_DefaultB_IPL_1 was identified in the left default B network. From Figure 9, it could be concluded that three groups of subjects showed left lateralization at 17Networks_LH_DefaultB_IPL_1 for LDegreeR and LStrengthR, and the magnitude of lateralization was significantly larger in the RLS group than in other two groups. fMRI studies have revealed significant differences in the pattern of functional connectivity of the inferior parietal lobule (IPL) between the left and right hemispheres: the left IPL was mainly involved in tool use and language processing, whereas the right IPL primarily participated in spatial attention and mathematical cognition (Zhang and Li, 2014). In particular, the left IPL demonstrated functional and anatomical heterogeneity, encompassing higher cognitive functions such as numerical judgment and arithmetic (Göbel and Rushworth, 2004; Hubbard et al., 2005), reading (Turkeltaub et al., 2002), recognition memory (Henson et al., 1999; O'Connor et al., 2010), semantic processing (Chou et al., 2006; Raposo et al., 2006) and tool use (Ishibashi et al., 2011; Peeters et al., 2009). In addition, it was demonstrated that the function between the ventral posterior nucleus and the right IPL was significantly diminished in migraine without aura (MWoA; Qin et al., 2020), and that MWoA patients with long-term disease duration exhibited a reduction in regional homogeneity (ReHo) value in the IPL (Chen et al., 2022; Zhao et al., 2013). The findings of this study indicated that RLS might further promote the degree and connectivity strength of 17Networks_LH_DefaultB_IPL_1 to concentrate in the left hemisphere, which might be related to cognitive impairments in patients. In addition, 17Networks_RH_DefaultB_PFCd_3, was located in the dorsal prefrontal cortex (dPFC) of the right default B network. The results in Figure 9 showed that in both the NRLS group and the normal group, the LDegreeR and LStrengthR of 17Networks_RH_DefaultB_PFCd_3 both exhibited right lateralization, whereas the RLS group displayed the opposite lateralization trend. The dPFC was a core region for higher cognitive functions such as cognitive control, decision-making, and working memory. Related studies have shown that the right dPFC played a special role in processing memory (Jones et al., 2004) and was also involved in design cognition in ill-structured situations (Gilbert et al., 2010). In addition, RS-fMRI studies have shown that individuals with migraine exhibit variability in BOLD signaling, enhanced functional connectivity, and changes in dynamic functional connectivity in the dPFC region (Schwedt et al., 2015; Schwedt et al., 2014; Lim et al., 2021; Schramm et al., 2023). Our findings indicated that RLS resulted in a change in the lateralization direction of LDegreeR and LStrengthR in 17Networks_RH_DefaultB_PFCd_3. This suggested that the functional connectivity and network properties of this region had undergone alterations, which might be associated with cognitive functions associated with migraine.

17Networks_RH_SomMotA_15 and 17Networks_RH_SomMotA_16 were located in the right somatomotor A network. Figure 9 showed that for 17Networks_RH_SomMotA_15, the three groups exhibited right lateralization in LFunctionCorrR, LBetweennessCentralityR, and LStrengthR, with the NRLS group and control group showing a greater degree of lateralization than the RLS group. Supplementary Tables S2, S3 indicated that in the NRLS group, LFunctionCorrR and LStrengthR of 17Networks_RH_SomMotA_15 were positively correlated with disease duration, suggesting that the lateralization of these network attributes might increase as the disease progresses. In the RLS group, LBetweennessCentralityR of 17Networks_RH_SomMotA_15 was also positively correlated with disease duration, indicating that the mediating role of this region might increase as the disease progressed. In addition, Figure 9 demonstrated that in the NRLS group and healthy control group, both LDegreeR and LStrengthR of 17Networks_RH_SomMotA_16 exhibited right lateralization, whereas the RLS group showed left lateralization. Supplementary Table S3 revealed that in the RLS group, LDegreeR and LStrengthR of 17Networks_RH_SomMotA_16 were positively correlated with VAS, suggesting that as pain intensity increased, the lateralization of network connectivity attributes in 17Networks_RH_SomMotA_16 might also strengthen. Several studies have demonstrated significant asymmetry in the motor system (Amunts et al., 1996; Toga and Thompson, 2003; Dinomais et al., 2016). Further investigations revealed extensive hierarchical network asymmetry in motor regions at rest state, reflecting the right hemisphere’s dominance in spatially attentive predictive motor coding (Yan et al., 2012). Studies on migraine further showed that compared to healthy controls, some sensorimotor networks (especially primary somatosensory cortex and right premotor cortex) in MWoA exhibited reduced ReHo, degree centrality (DC), and amplitude of low-frequency fluctuations (ALFF). Furthermore, resting-state FC within the SMN network was also decreased in vestibular migraineurs compared to the healthy group (Schramm et al., 2023; Li et al., 2022). In summary, the results of this study suggested that RLS might affect the lateralization properties of 17Networks_RH_SomMotA_15 and 17Networks_RH_SomMotA_16. These findings provided further support for the research on the asymmetry of the brain’s motor system and offered potential insights into the neuro-mechanistic mechanisms underlying neurological disorders such as migraine.

## 5 Limitations and future directions

It should be noted that this study still had some limitations. Firstly, the sample size of this study was relatively small. This issue was addressed to some extent by using independent testing dataset from different acquisition sources and increasing the sample size through the splitting of the TR. In the future more relevant patients will be introduced to improve the credibility and accuracy of the findings. Then, this study only considered the lateralization of static functional connectivity. However, functional connectivity in the brain is dynamically changing, and functional connectivity patterns might vary at different time points. Consequently, future studies could adopt dynamic functional connectivity analysis methods to explore the changes in brain lateralization at different time points.

## 6 Conclusion

In this study, a three-classification model was developed using lateralization of functional connectivity and network topology to investigate the relationship and impact of RLS and migraine lateralization. The results demonstrated that the laterality indices and the constructed classification model effectively distinguished between migraine patients with and without RLS. The classification rate based on the laterality indices of brain regions was 0.8961, and the accuracy of independent sample testing was 0.8874. After expanding and reclassifying the samples, the classification accuracy further improved to 0.9103 and 0.9099, respectively. Furthermore, the third sample set demonstrated a classification accuracy rate of 0.8745. Additionally, 9 key brain regions with high discriminative power were identified, which exhibited different lateralization behaviors among the three groups. This study not only enhanced our understanding of the pathological mechanisms of RLS and migraine but also introduced a new non-invasive approach for exploring potential biomarker associated with RLS and migraine.

## Data Availability

The datasets presented in this study can be found in online repositories. The names of the repository/repositories and accession number(s) can be found at: the migraine dataset generated and/or analyzed during the current study are not publicly available due to the Regulations on Human Genetic Resources Management published by the Chinese government. The data of normal controls were obtained from a free public database which can be accessed at http://fcon_1000.projects.nitrc.org/fcpClassic/FcpTable.html and http://fcon_1000.projects.nitrc.org/indi/retro/cobre.html. Codes that support the results of this study are available from the corresponding authors on reasonable request.

## Footnotes

^1^http://fcon_1000.projects.nitrc.org/fcpClassic/FcpTable.html

^2^http://fcon_1000.projects.nitrc.org/indi/retro/cobre.html

## References

[ref1] Achard S. Bullmore E. (2007). Efficiency and cost of economical brain functional networks. PLoS Comput. Biol. 3:e17. doi: 10.1371/journal.pcbi.0030017, PMID: 17274684 PMC1794324

[ref2] Ailani J. (2014). Migraine and patent foramen ovale. Current Neurol. Neurosci. Reports 14:426. doi: 10.1007/s11910-013-0426-4 24402405

[ref3] Amunts K. Schlaug G. Schleicher A. Steinmetz H. Dabringhaus A. Roland P. E.. (1996). Asymmetry in the human motor cortex and handedness. NeuroImage 4, 216–222. doi: 10.1006/nimg.1996.0073 9345512

[ref4] Asanowicz D. Marzecová A. Jaśkowski P. Wolski P. (2012). Hemispheric asymmetry in the efficiency of attentional networks. Brain Cogn. 79, 117–128. doi: 10.1016/j.bandc.2012.02.014, PMID: 22475579

[ref5] Beraha E. Eggers J. Hindi Attar C. Gutwinski S. Schlagenhauf F. Stoy M.. (2012). Hemispheric asymmetry for affective stimulus processing in healthy subjects–a fMRI study. PLoS One 7:e46931. doi: 10.1371/journal.pone.0046931, PMID: 23056533 PMC3466188

[ref6] Caeyenberghs K. Leemans A. (2014). Hemispheric lateralization of topological organization in structural brain networks. Hum. Brain Mapp. 35, 4944–4957. doi: 10.1002/hbm.22524, PMID: 24706582 PMC6869817

[ref7] Cao W. Shen Y. Zhong J. Chen Z. Wang N. Yang J. (2022). The patent foramen ovale and migraine: associated mechanisms and perspectives from MRI evidence. Brain Sci. 12:941. doi: 10.3390/brainsci12070941, PMID: 35884747 PMC9313384

[ref8] Chen Z.-H. Cui Y.-L. Sun J.-T. Li Y.-T. Zhang C. Zhang Y.-M.. (2022). The brain structure and function abnormalities of migraineurs: a systematic review and neuroimaging meta-analysis. Front. Neurol. 13:1022793. doi: 10.3389/fneur.2022.1022793, PMID: 36419535 PMC9676357

[ref9] Chou T. L. Booth J. R. Bitan T. Burman D. D. Bigio J. D. Cone N. E.. (2006). Developmental and skill effects on the neural correlates of semantic processing to visually presented words. Hum. Brain Mapp. 27, 915–924. doi: 10.1002/hbm.20231, PMID: 16575838 PMC2615534

[ref10] Coppola G. Di Renzo A. Tinelli E. Di Lorenzo C. Scapeccia M. Parisi V.. (2018). Resting state connectivity between default mode network and insula encodes acute migraine headache. Cephalalgia 38, 846–854. doi: 10.1177/0333102417715230, PMID: 28605972

[ref11] Dang L. Chen C. Duan Q. Wang D. Du X. (2021). Relationship between non-headache symptoms and right to left shunt in episodic migraine. J. Clin Neurosci. 86, 38–44. doi: 10.1016/j.jocn.2021.01.004, PMID: 33775344

[ref12] Dinomais M. Chinier E. Richard I. Ricalens E. Aubé C. Ter Minassian A. (2016). Hemispheric asymmetry of supplementary motor area proper: a functional connectivity study of the motor network. Mot. Control. 20, 33–49. doi: 10.1123/mc.2014-0076, PMID: 26186228

[ref13] Duboc V. Dufourcq P. Blader P. Roussigné M. (2015). Asymmetry of the brain: development and implications. Annu. Rev. Genet. 49, 647–672. doi: 10.1146/annurev-genet-112414-055322 26442849

[ref14] Gilbert S. J. Zamenopoulos T. Alexiou K. Johnson J. H. (2010). Involvement of right dorsolateral prefrontal cortex in ill-structured design cognition: An fMRI study. Brain Res. 1312, 79–88. doi: 10.1016/j.brainres.2009.11.045, PMID: 19948156

[ref15] Göbel S. M. Rushworth M. F. (2004). Cognitive neuroscience: acting on numbers. Curr. Biol. 14, R517–R519. doi: 10.1016/j.cub.2004.06.042 15242633

[ref16] Gotts S. J. Jo H. J. Wallace G. L. Saad Z. S. Cox R. W. Martin A. (2013). Two distinct forms of functional lateralization in the human brain. Proc. Natl. Acad. Sci. 110, E3435–E3444. doi: 10.1073/pnas.1302581110 23959883 PMC3767540

[ref17] Güntürkün O. Ströckens F. Ocklenburg S. (2020). Brain lateralization: a comparative perspective. Physiol. Rev. 100, 1019–1063. doi: 10.1152/physrev.00006.2019 32233912

[ref18] Henson R. N. Rugg M. Shallice T. Josephs O. Dolan R. J. (1999). Recollection and familiarity in recognition memory: an event-related functional magnetic resonance imaging study. J. Neurosci. 19, 3962–3972. doi: 10.1523/JNEUROSCI.19-10-03962.1999, PMID: 10234026 PMC6782715

[ref19] Hubbard E. M. Piazza M. Pinel P. Dehaene S. (2005). Interactions between number and space in parietal cortex. Nat. Rev. Neurosci. 6, 435–448. doi: 10.1038/nrn1684 15928716

[ref20] Ishibashi R. Ralph M. A. L. Saito S. Pobric G. (2011). Different roles of lateral anterior temporal lobe and inferior parietal lobule in coding function and manipulation tool knowledge: evidence from an rTMS study. Neuropsychologia 49, 1128–1135. doi: 10.1016/j.neuropsychologia.2011.01.004, PMID: 21219917

[ref21] Jones C. R. Rosenkranz K. Rothwell J. C. Jahanshahi M. (2004). The right dorsolateral prefrontal cortex is essential in time reproduction: an investigation with repetitive transcranial magnetic stimulation. Exp. Brain Res. 158, 366–372. doi: 10.1007/s00221-004-1912-3 15365666

[ref22] Ke J. Yu Y. Zhang X. Su Y. Wang X. Hu S.. (2020). Functional alterations in the posterior insula and cerebellum in migraine without aura: a resting-state MRI study. Front. Behav. Neurosci. 14:567588. doi: 10.3389/fnbeh.2020.567588, PMID: 33132860 PMC7573354

[ref23] Kumar P. Kijima Y. West B. H. Tobis J. M. (2019). The connection between patent foramen ovale and migraine. Neuroimag. Clin. 29, 261–270. doi: 10.1016/j.nic.2019.01.006, PMID: 30926116

[ref24] Küper M. Rabe K. Holle D. Savidou I. Dommes P. Frings M.. (2013). Prevalence of cardiac right left shunts in migraine: a population-based case–control study. Neurol. Sci. 34, 205–208. doi: 10.1007/s10072-012-0986-0, PMID: 22367223

[ref25] Li D. Cui X. Yan T. Liu B. Zhang H. Xiang J.. (2021). Abnormal rich club organization in hemispheric white matter networks of ADHD. J. Atten. Disord. 25, 1215–1229. doi: 10.1177/1087054719892887, PMID: 31884863

[ref26] Li Z.-Y. Si L.-H. Shen B. Yang X. (2022). Altered brain network functional connectivity patterns in patients with vestibular migraine diagnosed according to the diagnostic criteria of the Bárány society and the international headache society. J. Neurol., 269, 3036–3036. doi: 10.1007/s00415-021-10868-0 PMC9119883 34792633

[ref27] Lim M. Jassar H. Kim D. J. Nascimento T. D. DaSilva A. F. (2021). Differential alteration of fMRI signal variability in the ascending trigeminal somatosensory and pain modulatory pathways in migraine. J. headache pain 22, 1–15. doi: 10.1186/s10194-020-01210-6 33413090 PMC7791681

[ref28] Ling Y. Wang M. Pan X. Zhao H. (2020). Clinical features of right-to-left shunt in the different subgroups of migraine. Brain Behav. 10:e01553. doi: 10.1002/brb3.1553, PMID: 32011802 PMC7066358

[ref29] Liu H. Stufflebeam S. M. Sepulcre J. Hedden T. Buckner R. L. (2009). Evidence from intrinsic activity that asymmetry of the human brain is controlled by multiple factors. Proc. Natl. Acad. Sci. 106, 20499–20503. doi: 10.1073/pnas.0908073106, PMID: 19918055 PMC2777963

[ref30] Moulton E. Becerra L. Maleki N. Pendse G. Tully S. Hargreaves R.. (2011). Painful heat reveals hyperexcitability of the temporal pole in interictal and ictal migraine states. Cereb. Cortex 21, 435–448. doi: 10.1093/cercor/bhq109, PMID: 20562317 PMC3020583

[ref31] Nie W. Zeng W. Yang J. Shi Y. Zhao L. Li Y.. (2021). Extraction and analysis of dynamic functional connectome patterns in migraine sufferers: a resting-state fMRI study. Computational Mathematical Methods Med. 2021, 1–14. doi: 10.1155/2021/6614520, PMID: 33959191 PMC8075661

[ref32] O'Connor A. R. Han S. Dobbins I. G. (2010). The inferior parietal lobule and recognition memory: expectancy violation or successful retrieval? J. Neurosci. 30, 2924–2934. doi: 10.1523/JNEUROSCI.4225-09.2010, PMID: 20181590 PMC2844718

[ref33] Ogawa S. Lee T.-M. Kay A. R. Tank D. W. (1990). Brain magnetic resonance imaging with contrast dependent on blood oxygenation. Proc. Natl. Acad. Sci. 87, 9868–9872. 2124706 10.1073/pnas.87.24.9868 PMC55275

[ref34] Olesen J. (2018). Headache classification committee of the international headache society (IHS) the international classification of headache disorders. Cephalalgia 38, 1–211. doi: 10.1177/0333102417738202 29368949

[ref35] Olson I. R. Plotzker A. Ezzyat Y. (2007). The enigmatic temporal pole: a review of findings on social and emotional processing. Brain 130, 1718–1731. doi: 10.1093/brain/awm052 17392317

[ref36] Peeters R. Simone L. Nelissen K. Fabbri-Destro M. Vanduffel W. Rizzolatti G.. (2009). The representation of tool use in humans and monkeys: common and uniquely human features. J. Neurosci. 29, 11523–11539. doi: 10.1523/JNEUROSCI.2040-09.2009, PMID: 19759300 PMC6665774

[ref37] Qin Z. Su J. He X. Zhu Q. Cui Y. Zhang J.. (2020). Altered resting-state functional connectivity between subregions in the thalamus and cortex in migraine without aura. Eur. J. Neurol. 27, 2233–2241. doi: 10.1111/ene.14411, PMID: 32562320

[ref38] Raposo A. Moss H. E. Stamatakis E. A. Tyler L. K. (2006). Repetition suppression and semantic enhancement: an investigation of the neural correlates of priming. Neuropsychologia 44, 2284–2295. doi: 10.1016/j.neuropsychologia.2006.05.017, PMID: 16806317

[ref39] Russo A. Silvestro M. Tessitore A. Tedeschi G. (2018). Advances in migraine neuroimaging and clinical utility: from the MRI to the bedside. Expert. Rev. Neurother. 18, 533–544. doi: 10.1080/14737175.2018.1486708, PMID: 29883214

[ref40] Russo A. Silvestro M. Tessitore A. Tedeschi G. (2019). Shedding light on migraine with aura: the clarifying role of advanced neuroimaging investigations. Expert. Rev. Neurother. 19, 739–750. doi: 10.1080/14737175.2019.1638252, PMID: 31267785

[ref41] Safiri S. Pourfathi H. Eagan A. Mansournia M. A. Khodayari M. T. Sullman M. J.. (2022). Global, regional, and national burden of migraine in 204 countries and territories, 1990 to 2019. Pain 163, e293–e309. doi: 10.1097/j.pain.0000000000002275, PMID: 34001771

[ref42] Schaefer A. Kong R. Gordon E. M. Laumann T. O. Zuo X.-N. Holmes A. J.. (2018). Local-global parcellation of the human cerebral cortex from intrinsic functional connectivity MRI. Cereb. Cortex 28, 3095–3114. doi: 10.1093/cercor/bhx179, PMID: 28981612 PMC6095216

[ref43] Schramm S. Börner C. Reichert M. Baum T. Zimmer C. Heinen F.. (2023). Functional magnetic resonance imaging in migraine: a systematic review. Cephalalgia 43:03331024221128278. doi: 10.1177/03331024221128278, PMID: 36751858

[ref44] Schwedt T. J. Chiang C. C. Chong C. D. Dodick D. W. (2015). Functional MRI of migraine. Lancet Neurol. 14, 81–91. doi: 10.1016/S1474-4422(14)70193-0, PMID: 25496899 PMC11318354

[ref45] Schwedt T. J. Chong C. D. Chiang C.-C. Baxter L. Schlaggar B. L. Dodick D. W. (2014). Enhanced pain-induced activity of pain-processing regions in a case-control study of episodic migraine. Cephalalgia 34, 947–958. doi: 10.1177/0333102414526069 24627432 PMC4163130

[ref46] Shi Y. Zeng W. Nie W. Yang J. (2020). Multi-channel hierarchy functional integration analysis between large-scale brain networks for migraine: An fMRI study. NeuroImage: Clin. 28:102462. doi: 10.1016/j.nicl.2020.102462, PMID: 33395958 PMC7575876

[ref47] Shu N. Liu Y. Duan Y. Li K. (2015). Hemispheric asymmetry of human brain anatomical network revealed by diffusion tensor tractography. Biomed. Res. Int. 2015, 1–11. doi: 10.1155/2015/908917, PMID: 26539535 PMC4619913

[ref48] Silvestro M. Tessitore A. Di Nardo F. Scotto di Clemente F. Trojsi F. Cirillo M.. (2022). Functional connectivity changes in complex migraine aura: beyond the visual network. Eur. J. Neurol. 29, 295–304. doi: 10.1111/ene.15061, PMID: 34382315 PMC9291958

[ref49] Steiner T. Stovner L. Jensen R. Uluduz D. Katsarava Z. (2020). Migraine remains second among the world’s causes of disability, and first among young women: findings from GBD2019. J. Headache Pain 21, 1–4. doi: 10.1186/s10194-019-1071-3 33267788 PMC7708887

[ref50] Sun Y. Chen Y. Collinson S. L. Bezerianos A. Sim K. (2017). Reduced hemispheric asymmetry of brain anatomical networks is linked to schizophrenia: a connectome study. Cereb. Cortex 27, 602–615. doi: 10.1093/cercor/bhv255, PMID: 26503264

[ref51] Teshome M. K. Najib K. Nwagbara C. C. Akinseye O. A. Ibebuogu U. N. (2020). Patent foramen ovale: a comprehensive review. Curr. Probl. Cardiol. 45:100392. doi: 10.1016/j.cpcardiol.2018.08.004 30327131

[ref52] Toga A. W. Thompson P. M. (2003). Mapping brain asymmetry. Nat. Rev. Neurosci. 4, 37–48. doi: 10.1038/nrn1009 12511860

[ref53] Turkeltaub P. E. Eden G. F. Jones K. M. Zeffiro T. A. (2002). Meta-analysis of the functional neuroanatomy of single-word reading: method and validation. NeuroImage 16, 765–780. doi: 10.1006/nimg.2002.1131, PMID: 12169260

[ref54] Vincent M. Pedra E. Mourao-Miranda J. Bramati I. Henrique A. Moll J. (2003). Enhanced interictal responsiveness of the migraineous visual cortex to incongruent bar stimulation: a functional MRI visual activation study. Cephalalgia 23, 860–868. doi: 10.1046/j.1468-2982.2003.00609.x, PMID: 14616927

[ref55] Wahl A. Praz F. Tai T. Findling O. Walpoth N. Nedeltchev K.. (2010). Improvement of migraine headaches after percutaneous closure of patent foramen ovale for secondary prevention of paradoxical embolism. Heart 96, 967–973. doi: 10.1136/hrt.2009.181156, PMID: 20538672

[ref56] Waldstein S. R. Kop W. J. Schmidt L. A. Haufler A. J. Krantz D. S. Fox N. A. (2000). Frontal electrocortical and cardiovascular reactivity during happiness and anger. Biol. Psychol. 55, 3–23. doi: 10.1016/S0301-0511(00)00065-X, PMID: 11099805

[ref57] Wang B. Yang L. Yan W. An W. Xiang J. Li D. (2023). Brain asymmetry: a novel perspective on hemispheric network. Brain Sci. Advan. 9, 56–77. doi: 10.26599/BSA.2023.9050014

[ref58] Wilmshurst P. (2018). Migraine with aura and persistent foramen ovale. Eye 32, 184–188. doi: 10.1038/eye.2017.269, PMID: 29219954 PMC5811737

[ref59] Yan X. Kong R. Xue A. Yang Q. Orban C. An L.. (2023). Homotopic local-global parcellation of the human cerebral cortex from resting-state functional connectivity. NeuroImage 273:120010. doi: 10.1016/j.neuroimage.2023.120010, PMID: 36918136 PMC10212507

[ref60] Yan L.-R. WU Y.-B. HU D.-W. QIN S.-Z. XU G.-Z. ZENG X.-H.. (2012). Network asymmetry of motor areas revealed by resting-state functional magnetic resonance imaging. Behav. Brain Res. 227, 125–133. doi: 10.1016/j.bbr.2011.11.012, PMID: 22108343

[ref61] Yan C. Zang Y. (2010). DPARSF: a MATLAB toolbox for" pipeline" data analysis of resting-state fMRI. Front. Syst. Neurosci. 13:013. doi: 10.3389/fnsys.2010.00013 PMC2889691 20577591

[ref62] Yeo B. T. Krienen F. M. Sepulcre J. Sabuncu M. R. Lashkari D. Hollinshead M.. (2011). The organization of the human cerebral cortex estimated by intrinsic functional connectivity. J. Neurophysiol. 106:1125–65. doi: 10.1152/jn.00338.2011 21653723 PMC3174820

[ref63] Zhang S. Li C.-S. R. (2014). Functional clustering of the human inferior parietal lobule by whole-brain connectivity mapping of resting-state functional magnetic resonance imaging signals. Brain Connect. 4, 53–69. doi: 10.1089/brain.2013.0191, PMID: 24308753 PMC3929139

[ref64] Zhao L. Liu J. Dong X. Peng Y. Yuan K. Wu F.. (2013). Alterations in regional homogeneity assessed by fMRI in patients with migraine without aura stratified by disease duration. J. Headache Pain 14, 1–9. doi: 10.1186/1129-2377-14-85 24134520 PMC3853130

