# Supplementary material for: A three-classification model for identifying migraine with right-to-left shunt using lateralization of functional connectivity and brain network topology: a resting-state fMRI study
Source: Front Neurosci. 2024 Nov 12;18:1488193. doi: 10.3389/fnins.2024.1488193 (PMC11588730; doi:10.3389/fnins.2024.1488193)
Supplement: Supplementary file 1 [file Data_Sheet_1.docx]

Supplementary Material

# Supplementary Tables

# Table S1. Network metrics used in this study.

| **Network metric** | **Description** | **Definition** | **Explanation** |
| --- | --- | --- | --- |
| Betweenness Centrality | The fraction of all shortest paths in the network that contain a given node | $B_{i}=\frac{1}{\left( n-1 \right)*(n-2)}\sum_{\begin{aligned} h,j\epsilon N \\ h\neq j,j\neq i,h\neq i \end{aligned}} \frac{\rho_{hj}(i)}{\rho_{hj}}$ | N is the set of all nodes in the network; $\rho_{hj}$ is the number of shortest paths between h and j, and $\rho_{hj}$(i) is the number of shortest paths between h and j that pass through i. |
| Degree | Number of links connected to a node | $K_{i}=\sum_{j\epsilon N} a_{ij}$ | N is the set of all nodes in the network; (i, j) is a link between nodes i and j, (i, j $\epsilon$ N); and $a_{ij}$ is the status of the connection between i and j. |
| Strength | The sum of weights of links connected to a node | $S_{i}=\sum_{j\epsilon N} w_{ij}$ | N is the set of all nodes in the network; (i, j) is a link between nodes i and j, (i, j $\epsilon$ N); and $w_{ij}$ is the weighted connection value between i and j. |

# Table S2. The correlation coefficients and p-values between the lateralization of brain areas and clinical information for the NRLS group.

| **Laterality indices** | **ROI number** | **Duration**  **of illness** | **Frequency**  **of attacks** | **VAS** |
| --- | --- | --- | --- | --- |
| LFunctionCorrR | 135 | **-0.5373** | 0.2803 | 0.0169 |
|  |  | **0.0319** | 0.2930 | 0.9505 |
|  | 238 | **0.5031** | -0.3759 | 0.0067 |
|  |  | **0.0470** | 0.1513 | 0.9803 |
| LBetweenness  CentralityR | 238 | 0.2766 | -0.1880 | 0.2732 |
|  |  | 0.2997 | 0.4857 | 0.3059 |
| LDegreeR | 116 | -0.3217 | -0.0573 | 0.3259 |
|  |  | 0.2244 | 0.8330 | 0.2180 |
|  | 173 | 0.1599 | -0.3015 | -0.0675 |
|  |  | 0.5543 | 0.2564 | 0.8039 |
|  | 336 | 0.3624 | 0.3056 | 0.4448 |
|  |  | 0.1678 | 0.2498 | 0.0843 |
|  | 239 | -0.0259 | 0.0055 | -0.2350 |
|  |  | 0.9243 | 0.9839 | 0.3810 |
|  | 379 | 0.0368 | -0.2609 | -0.0972 |
|  |  | 0.8923 | 0.3292 | 0.7202 |
|  | 305 | 0.1098 | -0.0376 | -0.0145 |
|  |  | 0.6856 | 0.8901 | 0.9574 |
|  | 161 | -0.3260 | 0.1477 | -0.2453 |
|  |  | 0.2179 | 0.5853 | 0.3599 |
|  | 14 | -0.2933 | -0.0048 | 0.1700 |
|  |  | 0.2702 | 0.9858 | 0.5290 |
| LStrengthR | 116 | -0.2920 | -0.0983 | 0.3139 |
|  |  | 0.2725 | 0.7173 | 0.2364 |
|  | 336 | 0.3605 | 0.3138 | 0.4500 |
|  |  | 0.1701 | 0.2366 | 0.0803 |
|  | 173 | 0.1704 | -0.3201 | -0.0739 |
|  |  | 0.5281 | 0.2268 | 0.7856 |
|  | 379 | 0.0355 | -0.2573 | -0.0876 |
|  |  | 0.8963 | 0.3360 | 0.7469 |
|  | 14 | -0.2922 | -0.0439 | 0.1627 |
|  |  | 0.2720 | 0.8716 | 0.5472 |
|  | 238 | **0.5162** | -0.4114 | 0.1820 |
|  |  | **0.0407** | 0.1134 | 0.5000 |
|  | 239 | -0.0533 | 0.0031 | -0.2300 |
|  |  | 0.8445 | 0.9909 | 0.3914 |
|  | 161 | -0.3505 | 0.1073 | -0.2140 |
|  |  | 0.1832 | 0.6924 | 0.4261 |
|  | 135 | -0.1420 | 0.2046 | -0.1207 |
|  |  | 0.5999 | 0.4473 | 0.6561 |
|  | 305 | 0.1164 | -0.0384 | -0.0265 |
|  |  | 0.6678 | 0.8878 | 0.9224 |

# Table S3. The correlation coefficients and p-values between the lateralization of brain areas and clinical information for the RLS group.

| **Laterality indices** | **ROI number** | **Duration**  **of illness** | **Frequency**  **of attacks** | **VAS** | **RLS Grading** |
| --- | --- | --- | --- | --- | --- |
| LFunctionCorrR | 135 | 0.2857 | 0.2624 | **0.6338** | -0.4752 |
|  |  | 0.4235 | 0.4640 | **0.0491** | 0.1651 |
|  | 238 | 0.0932 | -0.3786 | 0.4671 | -0.0119 |
|  |  | 0.7978 | 0.2807 | 0.1735 | 0.9740 |
| LBetweenness  CentralityR | 238 | **0.7397** | 0.5169 | 0.5693 | -0.3015 |
|  |  | **0.0145** | 0.1260 | 0.0858 | 0.3972 |
| LDegreeR | 116 | -0.1468 | -0.1587 | -0.2736 | -0.0161 |
|  |  | 0.6858 | 0.6615 | 0.4442 | 0.9649 |
|  | 173 | 0.3486 | -0.0453 | 0.5260 | 0.0811 |
|  |  | 0.3236 | 0.9012 | 0.1183 | 0.8237 |
|  | 336 | 0.1919 | -0.0920 | 0.1253 | 0.0484 |
|  |  | 0.5953 | 0.8004 | 0.7301 | 0.8943 |
|  | 239 | -0.0402 | -0.2801 | **0.6608** | 0.3234 |
|  |  | 0.9122 | 0.4332 | **0.0375** | 0.3621 |
|  | 379 | 0.1808 | 0.1056 | -0.1702 | -0.0121 |
|  |  | 0.6172 | 0.7716 | 0.6383 | 0.9735 |
|  | 305 | -0.5527 | -0.1526 | 0.1016 | -0.1215 |
|  |  | 0.0976 | 0.6738 | 0.7800 | 0.7381 |
|  | 161 | -0.1433 | 0.3223 | 0.0163 | -0.4338 |
|  |  | 0.6929 | 0.3638 | 0.9643 | 0.2103 |
|  | 14 | -0.1471 | -0.2151 | -0.4931 | -0.1475 |
|  |  | 0.6852 | 0.5507 | 0.1475 | 0.6843 |
| LStrengthR | 116 | -0.1285 | -0.1473 | -0.2771 | -0.0343 |
|  |  | 0.7236 | 0.6848 | 0.4383 | 0.9250 |
|  | 336 | 0.2043 | -0.1119 | 0.1065 | 0.0297 |
|  |  | 0.5713 | 0.7582 | 0.7697 | 0.9351 |
|  | 173 | 0.3410 | -0.0191 | 0.5169 | 0.1100 |
|  |  | 0.3350 | 0.9581 | 0.1260 | 0.7623 |
|  | 379 | 0.1785 | 0.0974 | -0.1778 | -0.0272 |
|  |  | 0.6218 | 0.7889 | 0.6231 | 0.9406 |
|  | 14 | -0.1481 | -0.1871 | -0.4830 | -0.1482 |
|  |  | 0.6830 | 0.6048 | 0.1573 | 0.6828 |
|  | 238 | -0.0845 | -0.1849 | 0.6164 | 0.1528 |
|  |  | 0.8166 | 0.6091 | 0.0577 | 0.6734 |
|  | 239 | -0.0338 | -0.2695 | **0.6725** | 0.3129 |
|  |  | 0.9261 | 0.4514 | **0.0331** | 0.3787 |
|  | 161 | -0.1084 | 0.3628 | -0.0213 | -0.5144 |
|  |  | 0.7656 | 0.3029 | 0.9534 | 0.1282 |
|  | 135 | 0.2411 | 0.2720 | 0.3586 | **-0.7587** |
|  |  | 0.5023 | 0.4471 | 0.3089 | **0.0110** |
|  | 305 | -0.5474 | -0.1713 | 0.1064 | -0.0973 |
|  |  | 0.1015 | 0.6360 | 0.7698 | 0.7892 |

# Supplementary Figure


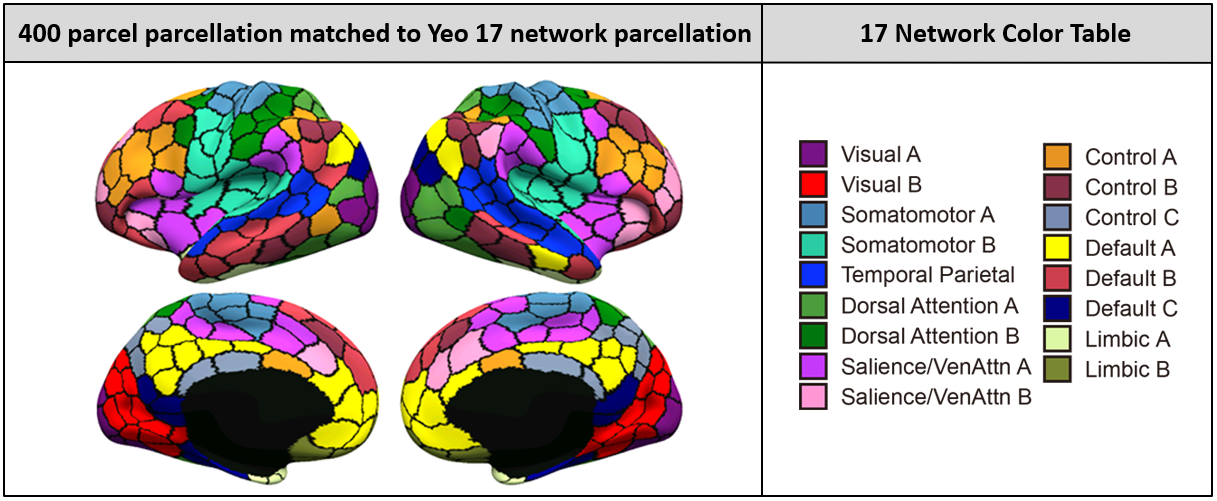


**Fig. S1.** **The visualization of the 400 parcel which were colored to match 400 brain areas.**


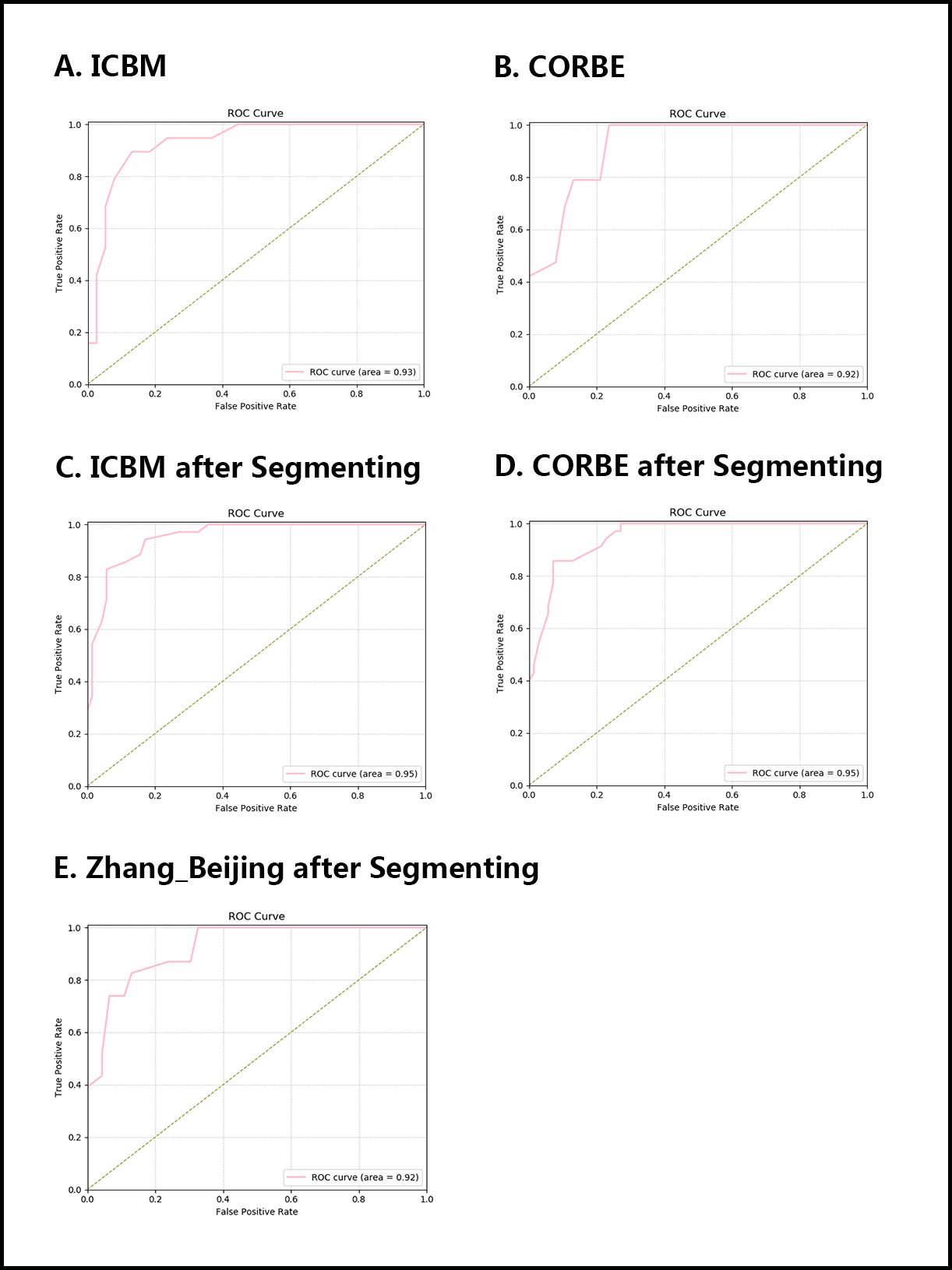


**Fig. S2.** **The ROC curve for all features classification.**

**
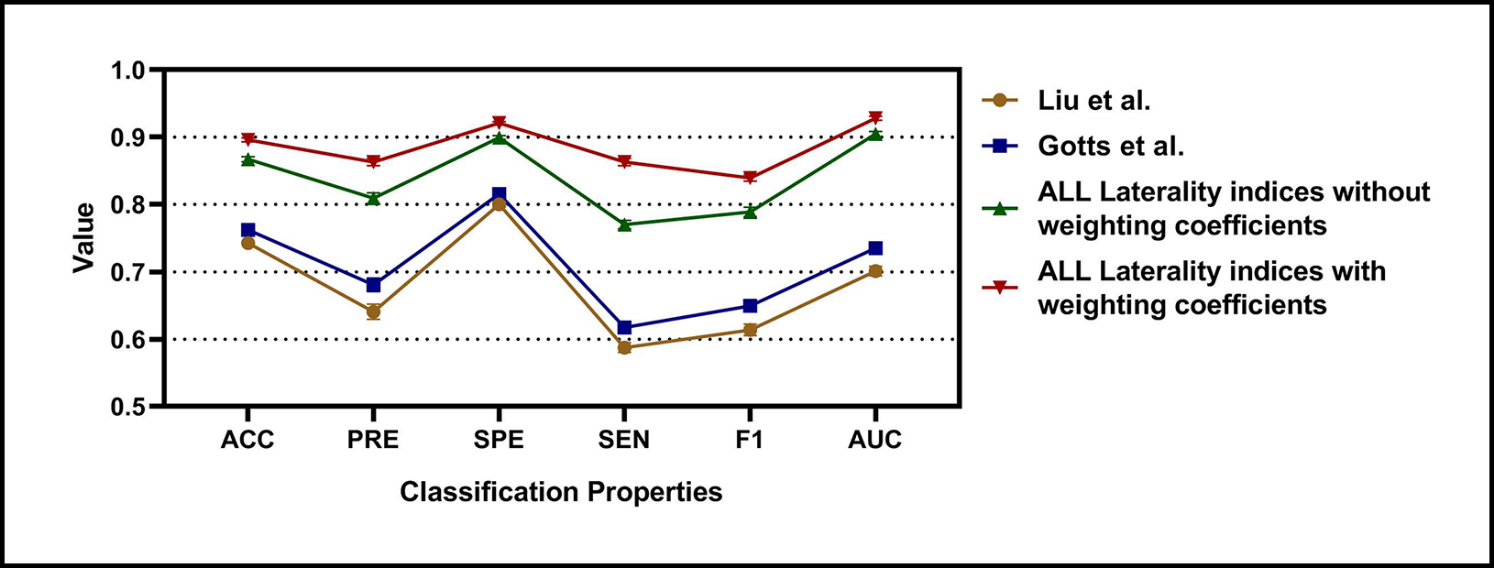
**

**Fig. S3. The classification Accuracy, Precision, Specificity, Sensitivity, F1, and AUC for different approaches for RLS, NRLS and ICBM groups.**

**
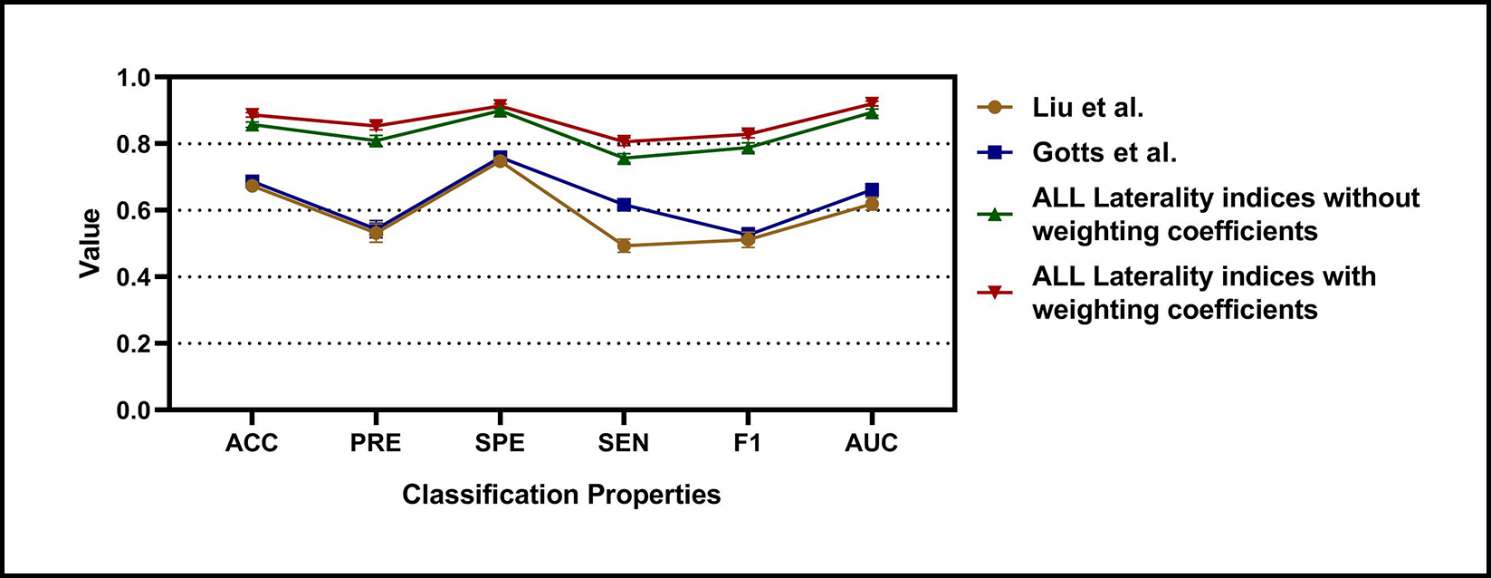
**

**Fig. S4. The classification Accuracy, Precision, Specificity, Sensitivity, F1, and AUC for different approaches for RLS, NRLS and COBRE groups.**
